# Temporally programmed STING nanoadjuvant delivery unlocks synergistic chemotherapy-induced antitumor immunity

**DOI:** 10.1126/sciadv.adw0797

**Published:** 2025-07-18

**Authors:** Zimeng Yang, Hengzhi Liu, Shuo Li, Zhaochu Xu, Wenxiao Li, Yubo Liu, Qingzhi Lv, Hongzhuo Liu, Zhonggui He, Yongjun Wang

**Affiliations:** ^1^ Wuya College of Innovation, Shenyang Pharmaceutical University, Shenyang, Liaoning 110016, China.; ^2^ School of Pharmacy, Binzhou Medical University, Yantai 264003, China.; ^3^ Joint International Research Laboratory of Intelligent Drug Delivery Systems, Ministry of Education, Shenyang Pharmaceutical University, Shenyang, Liaoning 110016, China.

## Abstract

Stimulator of interferon genes (STING) agonists have attracted notable attention for their potent immune activation capabilities. However, their clinical application is hindered by systemic toxicity and delivery inefficiencies. We addressed these challenges by developing a lymph node–targeted STING agonist nanoadjuvant (Mn/MSA-2@Lipo) combined with a temporally optimized delivery strategy. Mn/MSA-2@Lipo uses manganese ions to amplify STING pathway activation while achieving efficient lymph node accumulation and antigen presentation. We first induced immunogenic cell death (ICD) through chemotherapy to release tumor antigens, followed by the administration of the nanoadjuvant at an optimized time interval, the approach effectively synchronizes dendritic cell (DC) antigen uptake and maturation. This combination therapy notably enhanced antitumor immunity in melanoma and breast cancer models, achieving complete tumor regression and inducing long-lasting immune memory, all while demonstrating an excellent safety profile. Our findings highlight the critical importance of delivery timing optimization, offering a promising strategy for the clinical translation of STING agonists and the design of advanced immunotherapies.

## Teaser

Temporally programmed STING nanodelivery enhances chemoimmunotherapy, driving durable tumor control with reduced toxicity.

## INTRODUCTION

Cancer immunotherapy has revolutionized the landscape of oncology by harnessing the immune system to combat tumors. Central to its efficacy is the development of adjuvants that bridge innate and adaptive immunity (*1*). Among these, stimulator of interferon genes (STING) agonists have garnered particular interest due to their ability to initiate robust type I interferon (IFN-I) response, thereby stimulating immune cell activation and infiltration within the tumor microenvironment (TME) (*2*, *3*). Despite their promise, several critical factors have constrained the clinical translation of STING agonists, including systemic toxicity, poor bioavailability, and suboptimal delivery to immune-rich sites like lymph nodes, where effective immune activation is paramount (*4*, *5*). Addressing these issues is critical for unlocking the full therapeutic potential of STING agonists.

Lymph nodes play a pivotal role in orchestrating adaptive immunity as the primary sites for antigen presentation and T cell priming (*6*–*9*). Effective delivery of STING agonists to lymph nodes could substantially enhance therapeutic outcomes, but current delivery methods often fail to achieve this goal (*10*). For example, MSA-2, a high-affinity human-STING agonist, exhibits low oral bioavailability, necessitating impractically high doses (e.g., 80 mg/kg in mice) to achieve therapeutic effects (*11*). Alternative administration routes, such as systemic or subcutaneous delivery, are limited by poor intracellular uptake and rapid systemic clearance (*12*). These limitations not only reduce the efficacy of STING agonists but also restrict their ability to fully activate the immune system, particularly within key immunological sites like lymph nodes.

Improving the delivery of STING agonists can partially address these challenges, but standalone delivery solutions may not fully resolve the underlying issues. For instance, even with efficient lymph node targeting, insufficient antigen availability can limit immune activation. This highlights the potential of combining STING agonists with complementary therapies that can enhance antigen release and immune priming. Chemotherapy, in particular, offers a compelling solution by inducing immunogenic cell death (ICD), which releases tumor-associated antigens (TAAs) and creates a pro-inflammatory environment that synergizes with STING-mediated immune activation (*13*–*15*). For example, platinum-based chemotherapeutics conjugated with MSA-2 have shown synergistic antitumor effects in preclinical studies (*16*, *17*). In addition, researchers have developed nanoparticle-based delivery systems to improve the biodistribution and therapeutic efficacy of MSA-2. A recent study designed and used a supramolecular nanovector HCCSM for in situ vaccination immunotherapy of colorectal cancer (*18*, *19*). Other approaches, such as polymeric pro-agonists (*20*, *21*), iron-based nanoadjuvants (*22*, *23*), and mesoporous polydopamine platforms (*24*), have demonstrated enhanced lymphatic targeting, reduced systemic toxicity, and notable tumor suppression when combined with MSA-2. These approaches highlight the potential of integrating STING agonists into multifaceted treatment regimens.

Despite these advances, most current strategies rely on encapsulating multiple therapeutic agents within a single nanocarrier for simultaneous or sequential release. Although effective in some cases, this “one-size-fits-all” strategy often overlooks the advantages of precisely timed delivery, where synchronizing immune activation with antigen availability can lead to superior therapeutic outcomes. As a result, the full therapeutic potential of each component may not be realized.

To bridge these critical gaps, we propose a strategy that integrates a lymph node–targeted STING agonist nanoadjuvant with a temporally programmed delivery approach. Specifically, we developed Mn/MSA-2@Lipo, a liposome-based carrier for MSA-2 that is administered subcutaneously and incorporates Mn^2+^ to amplify STING pathway activation (*25*, *26*). By administering chemotherapy first to induce ICD and release TAAs, followed by the delayed administration of Mn/MSA-2@Lipo, this approach synchronizes immune activation with the peak antigen availability, enhancing dendritic cell (DC) activation and cross-presentation to T cells.

This strategy not only overcomes the limitations of current STING agonist therapies but also underscores the transformative potential of harnessing spatiotemporal precision to effectively counter immune suppression within the TME. By simultaneously enhancing therapeutic efficacy and minimizing systemic toxicity, this approach establishes a robust foundation for advancing combination cancer immunotherapy. Furthermore, it marks a notable milestone in the clinical translation of STING agonists, offering a promising pathway toward more effective and broadly applicable immunotherapeutic strategies.

## RESULTS

### Mn/MSA-2@Lipo demonstrates excellent physicochemical properties and lymphatic targeting potential

**Fig. 1.**
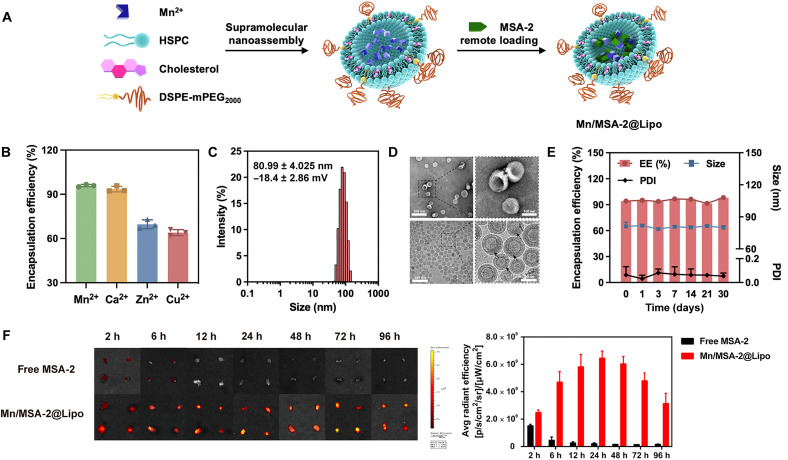
Liposome-based nanoadjuvant encapsulating MSA-2 for lymph node–targeted delivery. (**A**) Schematic illustrates the supramolecular nanoassembly structure formed by multiple lipid components, followed by remote loading of MSA-2 into the liposome interior. (**B**) EE of MSA-2 in liposomes prepared with various trapping agents. (**C**) Particle size distribution and zeta potential of Mn/MSA-2@Lipo. (**D**) Representative TEM (top) and cryo-TEM (bottom) images of Mn/MSA-2@Lipo, with Mn^2+^-MSA-2 complexes indicated by arrows. (**E**) Stability of Mn/MSA-2@Lipo stored at 4°C, evaluated by monitoring the particle size, PDI, and EE over 30 days. (**F**) Fluorescence imaging and quantitative analysis of axillary and inguinal lymph nodes following subcutaneous injection of free MSA-2 and Mn/MSA-2@Lipo. h, hours. Data are presented as means ± SD (*n* = 3).

We developed a scalable, liposome-based nanoadjuvant with high drug-loading capacity and encapsulation efficiency (EE) by leveraging coordination bonding between metal ions and carboxyl groups (Fig. 1A) (*26*, *27*). Briefly, we mixed an ethanol solution containing lipids with aqueous solutions of various metal ions, followed by extrusion through a polycarbonate membrane. After removing unincorporated metal ions, we incubated the liposomes with MSA-2 at 60°C to facilitate remote loading driven by the metal ion gradient. Among the tested metal ions, Mn^2+^ and Ca^2+^ exhibited the highest loading efficiencies (95.9 ± 0.8% and 92.5 ± 1.9%, respectively; Fig. 1B). Given that Ca^2+^ does not activate the STING pathway, we used Ca/MSA-2@Lipo as a negative control to highlight the STING-activating role of Mn^2+^. Mn/MSA-2@Lipo showed favorable physicochemical properties, with a hydrodynamic diameter of 80.99 ± 4.025 nm and a low polydispersity index (PDI) of 0.064 ± 0.069 (Fig. 1C). The formulation had a drug-loading capacity of 16%, indicating high uniformity and efficiency. Transmission electron microscopy (TEM) and cryo-TEM images revealed uniform bilamellar liposomes, likely formed due to osmotic pressure differences between the internal and external aqueous phases during drug loading (*28*), which caused deformation and invagination of monolayer liposomes into bilayers (Fig. 1E). Complexes of metal ions and MSA-2 were observed within the spherical interior of the liposomes (indicated by arrows).

We evaluated the release profiles of Mn/MSA-2@Lipo in both phosphate-buffered saline (PBS) and biologically relevant environments. The nanoadjuvant exhibited substantially higher cumulative release rates at pH 5.5 than at pH 7.4, suggesting efficient intracellular release of both Mn^2+^ and MSA-2 within lysosomes. This pH-responsive profile ensures synchronized delivery after DC internalization, optimizing STING pathway activation (fig. S1). Mn/MSA-2@Lipo also demonstrated excellent stability, maintaining consistent particle size and EE with no detectable drug leakage for 30 days at 4°C. It remained stable for up to 3 days under physiological conditions (Fig. 1D and fig. S2).

Efficient delivery of STING agonists to lymph nodes is critical for maximizing antitumor immunity and minimizing off-target toxicity. To assess lymphatic targeting, we used the IVIS imaging system to monitor the accumulation of free MSA-2 or Mn/MSA-2@Lipo following subcutaneous injection at the tail base of C57BL/6 mice. MSA-2’s intrinsic fluorescence (Ex = 405 nm, Em = 450 nm) provided a reliable readout, and metal ion trapping agents did not interfere with its signal (fig. S3). Free MSA-2 exhibited low and transient accumulation in lymph nodes, peaking at 2 hours postinjection and dissipating quickly. In contrast, Mn/MSA-2@Lipo reached peak accumulation at 6 hours and retained detectable signals for up to 72 hours. Quantification by inductively coupled plasma mass spectrometry (ICP-MS) and liquid chromatography–mass spectrometry (LC-MS) confirmed these results, showing high levels of Mn^2+^ and MSA-2 in draining lymph nodes from 12 to 48 hours postinjection (fig. S4). Two features likely contributed to this effective lymphatic targeting: (i) a particle size of ~80 nm, which facilitates transport through the subcutaneous matrix into lymphatic vessels while avoiding rapid absorption into blood capillaries (*29*, *30*), and (ii) a hydrophilic polyethylene glycol (PEG) coating, which reduces protein adsorption and promotes lymphatic system interaction (*31*, *32*). These design elements ensure high local drug concentrations in lymph nodes, reduce systemic exposure, and improve the safety profile of STING agonist-based therapies.

### Mn^2+^ enhances STING pathway activation and DC maturation via liposomal MSA-2 delivery

**Fig. 2.**
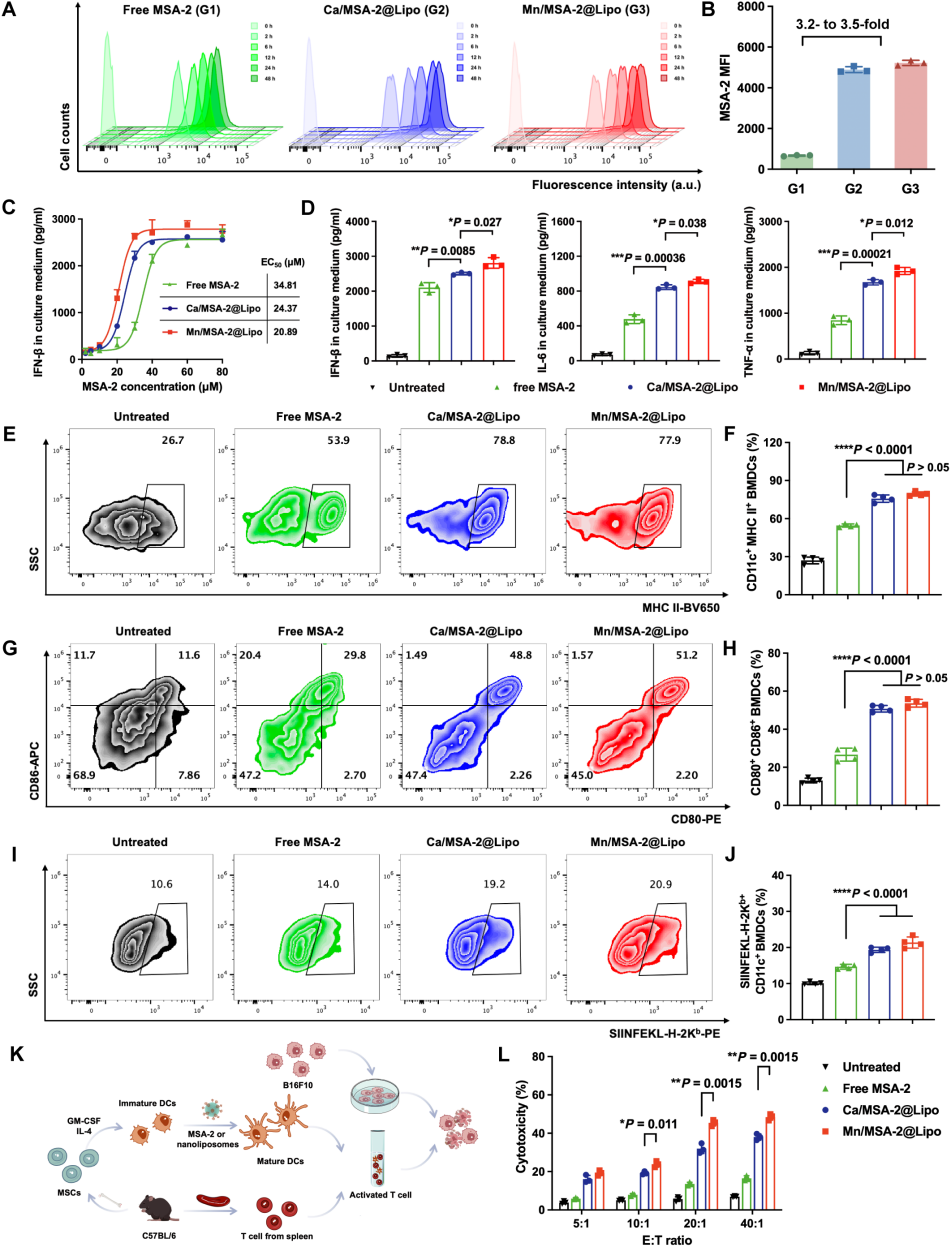
Enhanced internalization of MSA-2 and activation of DCs by MSA-2-loaded nanoliposomes in vitro. Flow cytometry analysis (**A**) and MFI (**B**) of BMDCs at various time points following treatment with different formulations. (**C**) Dose-response curves of IFN-β secretion by BMDCs treated with various formulations, measured in culture supernatants using ELISA. (**D**) Concentrations of IFN-β, IL-6, and TNF-α in BMDC culture supernatants after treatment with 40 μM MSA-2. Activation markers of BMDCs were significantly up-regulated after treatment with Mn/MSA-2@Lipo, including MHC II (**E** and **F**) and costimulatory molecules CD80/CD86 (**G** and **H**). (**I** and **J**) Mn/MSA-2@Lipo enhanced antigen cross-presentation by BMDCs, as indicated by the percentage of cells presenting OVA peptide (OVA_257-264_, SIINFEKL), determined by flow cytometry. (**K**) Combined incubation of OVA peptide and Mn/MSA-2@Lipo increased cytotoxic T lymphocyte–mediated lysis of B16F10-OVA cancer cells in vitro. (**L**) Cytolytic activity was quantified using LDH release assay at different effector-to-target cell (E:T) ratios (5:1, 10:1, 20:1, and 40:1). Data are presented as means ± SD (*n* = 4). Statistical significance was determined by one-way ANOVA with Tukey’s post hoc test. *P < 0.05, **P < 0.01, ***P < 0.001, and ****P < 0.0001.

The bilayer phospholipid structure of liposomes mimics cellular membranes, enabling direct fusion or endocytic uptake by immune cells such as DCs (*33*). To assess intracellular delivery, murine bone marrow–derived dendritic cells (BMDCs) were incubated with either free MSA-2 or liposome-encapsulated MSA-2. Fluorescence imaging revealed markedly higher intracellular accumulation of MSA-2 when delivered via liposomes, indicating enhanced uptake (Fig. 2, A and B). Subcellular localization studies further demonstrated successful lysosomal escape of MSA-2, facilitating cytosolic delivery required for STING activation (fig. S5).

We next evaluated inflammatory signaling by stimulating BMDCs in vitro with various concentrations of free MSA-2 or liposomal formulations. Liposomal MSA-2 induced substantially greater secretion of IFN-β than the free form, and inclusion of Mn^2+^ in the liposomes further enhanced this effect, achieving notable activity even at low median effective concentration (EC_50_) values (Fig. 2C). Moreover, levels of tumor necrosis factor–α (TNF-α) and interleukin-6 (IL-6) were also markedly elevated following Mn/MSA-2@Lipo treatment (Fig. 2D). We then validated STING pathway activation in human monocytic (THP-1) cells: Mn/MSA-2@Lipo maximally up-regulated IFN-β, TNF-α, and IL-6 secretion (fig. S6) and triggered robust phosphorylation of STING, TBK1, and IRF3, with levels rising 4.2-, 2.8-, and 2.7-fold, respectively, relative to untreated controls (fig. S7). In contrast, the nanoadjuvant failed to induce p-STING or IFN secretion in STING-knockout THP-1 cells, confirming that its effects are strictly STING-dependent (figs. S8 and S9). These findings demonstrate that liposomal encapsulation facilitates efficient intracellular delivery and that incorporating Mn^2+^ into the MSA-2 nanoliposome further enhances STING activation and downstream cytokine production, positioning Mn/MSA-2@Lipo as a potent amplifier of innate immune responses.

Cytokines such as IFN-β not only exert direct antitumor effects but also facilitate DC maturation and bridge innate and adaptive immunity (*34*). To evaluate this function, BMDCs were exposed to either free MSA-2 or liposomal formulations. Both Mn/MSA-2@Lipo and Ca/MSA-2@Lipo markedly up-regulated major histocompatibility complex class II (MHC II) and costimulatory molecules CD80 and CD86, whereas free MSA-2 had only a modest effect (Fig. 2, E to H). When these matured DCs were pulsed with the model antigen ovalbumin (OVA), those treated with Mn/MSA-2@Lipo exhibited a 1.5-fold increase in SIINFEKL-H-2K^b^ presentation compared to free MSA-2, indicating improved antigen cross-presentation (Fig. 2, I and J).

To determine the functional consequence of enhanced DC activation, we performed cytotoxicity assays using CD8^+^ T cells primed by treated BMDCs and B16F10 melanoma cells as targets. Mn/MSA-2@Lipo elicited the highest tumor cell lysis, with cytotoxicity levels markedly higher than those induced by Ca/MSA-2@Lipo or free MSA-2 (Fig. 2, K and L). T cells isolated directly from spleens showed negligible cytolytic activity, reinforcing the critical role of DC-mediated priming. These results underscore the synergistic effect of Mn^2+^ and MSA-2 codelivery on STING pathway activation and subsequent adaptive immune responses.

To validate these findings in vivo, tumor-free mice were administered Mn/MSA-2@Lipo, Mn^2+^@Lipo, Ca^2+^@Lipo, or free MSA-2 (25 mg/kg). Tumor-draining lymph nodes (tdLNs) were analyzed for DC maturation markers. Ca^2+^@Lipo failed to activate DCs, and Mn^2+^@Lipo or free MSA-2 produced only modest increases in CD80, CD86, and MHC II expression. In contrast, Mn/MSA-2@Lipo significantly increased the frequency of CD80^+^CD86^+^ and MHC II^+^ DCs (fig. S10). These results confirm that codelivery of Mn^2+^ and MSA-2 within a liposomal nanocarrier synergistically enhances lymph node immune activation, establishing Mn/MSA-2@Lipo as a promising nanoadjuvant for cancer immunotherapy.

### Combination therapy with chemotherapy and Mn/MSA-2@Lipo nanoadjuvant substantially enhances antitumor efficacy

**Fig. 3.**
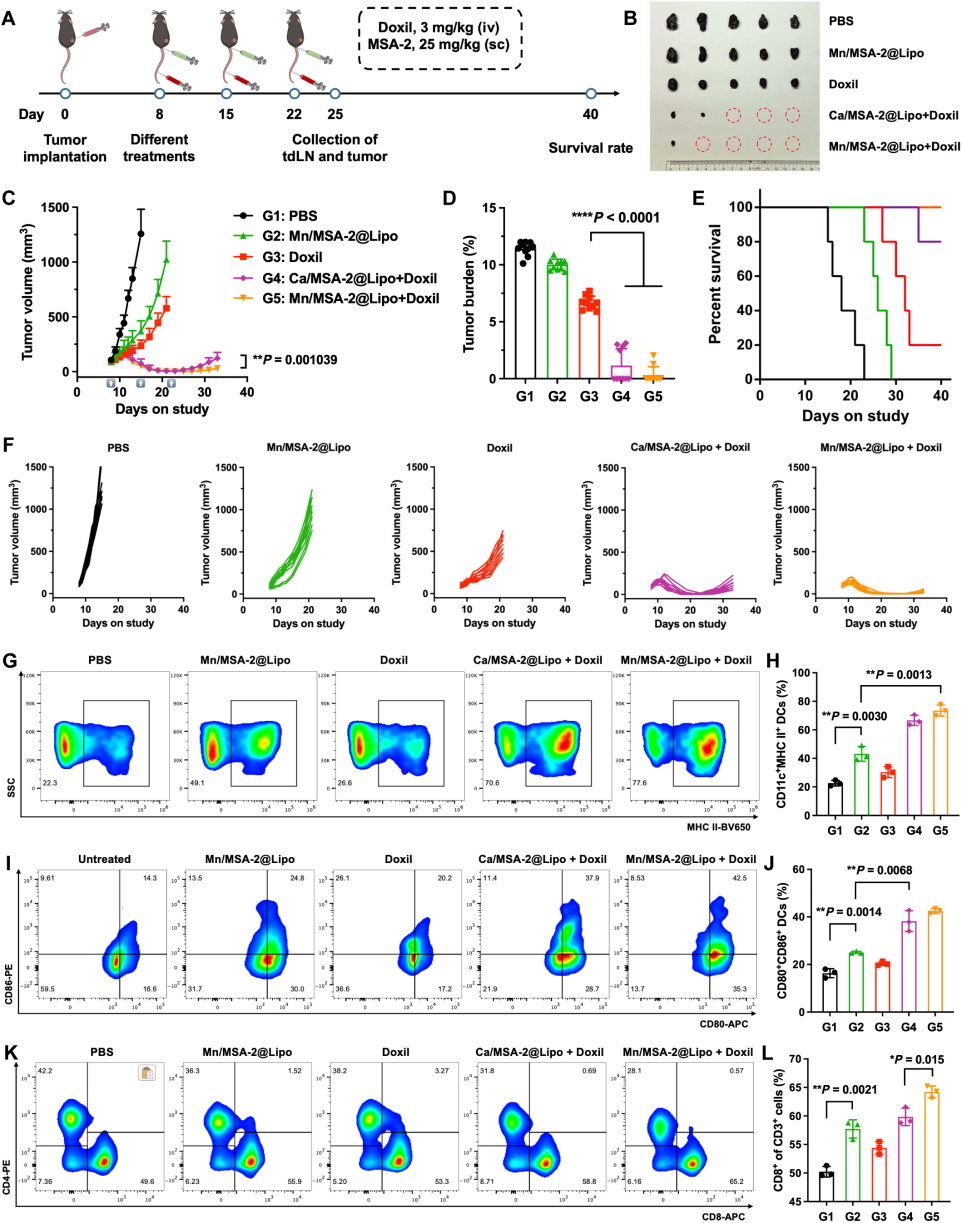
Combination therapy of chemotherapy and nanoadjuvants induces significant tumor regression in a B16F10 melanoma model. (**A**). Experimental design: B16F10 melanoma cells were subcutaneously inoculated into C57BL/6 mice. Tumor-bearing mice were treated with intravenous (iv) injections of Doxil (3 mg/kg) and subcutaneous (sc) injections of nanoadjuvants (25 mg/kg) on days 8, 15, and 22 postinoculation. (**B**) Representative images of B16F10 tumors on day 25 postinoculation. Tumor growth curve (**C**), tumor burden (**D**), and survival curve (**E**) of mice receiving different treatments. (**F**) Individual tumor growth curves for each B16F10 tumor-bearing mouse. Flow cytometry scatterplots and quantification of MHC II^+^ expression (**G** and **H**) and CD80^+^CD86^+^ (**I** and **J**) expression on DCs in tdLNs. Flow cytometry analysis of tumor-infiltrating CD4^+^ and CD8^+^ T cells in treated mice (**K** and **L**). Data are presented as means ± SD (*n* = 3). Statistical significance was determined by one-way ANOVA with Tukey’s post hoc test. *P < 0.05, **P < 0.01, and ****P < 0.0001.

Given the potent innate immune activation induced by Mn/MSA-2@Lipo, we next evaluated its antitumor efficacy in combination with chemotherapy. In a poorly immunogenic B16F10 melanoma model, 1 × 10^6^ cells were subcutaneously implanted into the right dorsal flank of C57BL/6 mice. Treatment commenced once tumors reached 60 to 80 mm^3^. Nanoadjuvants were administered subcutaneously to target lymph node, whereas the chemotherapeutic agent Doxil was delivered intravenously for systemic administration (Fig. 3A). The results showed that administration of Mn^2+^@Lipo, Ca^2+^@Lipo, or MSA-2 alone exhibited minimal antitumor efficacy. Monotherapy with Mn/MSA-2@Lipo (25 mg/kg) exhibited minimal antitumor effect, and Doxil alone (3 mg/kg) provided moderate efficacy. When combined with chemotherapy, the Ca^2+^@Lipo + Doxil group demonstrated antitumor effects comparable to Doxil alone, whereas the Mn^2+^@Lipo + Doxil group and MSA-2 + Doxil group achieved moderate tumor growth inhibition (fig. S11). However, the combination therapy significantly enhanced therapeutic outcomes, resulting in complete tumor regression in 60% (Ca/MSA-2@Lipo + Doxil) and 80% (Mn/MSA-2@Lipo + Doxil) of treated mice by day 21 (Fig. 3, B to F). Posttreatment monitoring revealed that the Mn/MSA-2@Lipo + Doxil group had the most prolonged tumor remission, likely due to the enhanced immune activation mediated by Mn/MSA-2@Lipo (Fig. 3, C and F). Safety assessments including body weight, hematoxylin and eosin (H&E) staining, and serum biochemistry revealed minimal systemic toxicity (figs. S12 to S14).

We next investigated the immunological mechanisms underlying this therapeutic synergy. Flow cytometry of tdLNs showed that, although Doxil alone did not significantly alter DC maturation, and nanoadjuvants alone induced only modest effects, the combination therapy substantially increased MHC II^+^ and CD80^+^CD86^+^ DCs. Mn/MSA-2@Lipo + Doxil produced the highest proportion of mature DCs in tdLNs (Fig. 3, G to J). Furthermore, the immune microenvironment was markedly improved, with a pronounced increase in CD8^+^ T cell infiltration and an elevated CD8^+^/CD4^+^ T cell ratio in the Mn^2+^-containing groups (Fig. 3, K and L, and fig. S15).

To confirm enhancement of antigen presentation in vivo, we used a B16F10-OVA tumor model and evaluated SIINFEKL-H-2K^b^ tetramer-positive CD8^+^ T cells. Although PBS-treated controls exhibited only 0.80 ± 0.09% H-2K^b^-specific CD8^+^ T cells, Mn/MSA-2@Lipo treatment increased this population to 16.2 ± 1.9% (figs. S16 and S17), consistent with in vitro results and indicating strong enhancement of cross-presentation by DCs. Together, these findings support that Mn^2+^ synergistically amplifies immune activation when codelivered with MSA-2 and chemotherapy.

**Fig. 4.**
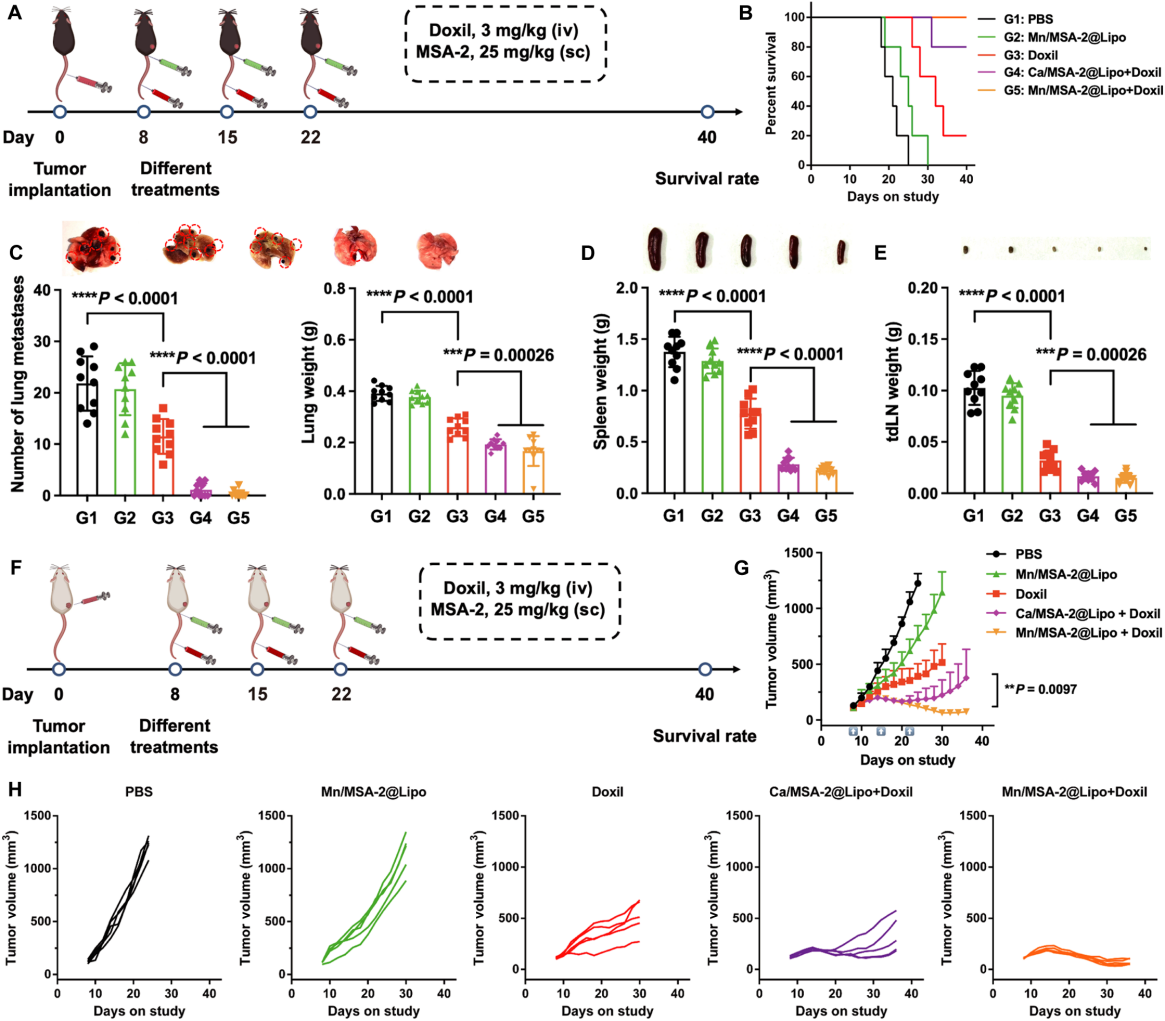
Combination therapy of chemotherapy and nanoadjuvants demonstrates therapeutic efficacy across different cancer models. (**A**) Experimental design: B16F10 melanoma cells were intravenously inoculated into C57BL/6 mice to establish a melanoma lung metastasis model. Tumor-bearing mice were treated with intravenous injections of Doxil (3 mg/kg) and subcutaneous injections of nanoadjuvants (25 mg/kg) on days 8, 15, and 22 postinoculation. (**B**) Survival curves of mice receiving different treatment regimens. (**C**) Quantification of lung metastatic nodules and lung weight in each treatment group. Spleen weights (**D**) and tdLN weights (**E**) for each treatment group. (**F**) Schematic representation of the treatment regimen and model construction for the 4T1 breast cancer model in Balb/c mice. (**G**) Tumor growth curve for mice treated with different regimens in the 4T1 breast cancer model. (**H**) Individual tumor growth curves for each 4T1 tumor-bearing mouse. Data are presented as means ± SD (*n* = 5). Statistical significance was determined by one-way ANOVA with Tukey’s post hoc test. **P < 0.01, ***P < 0.001, and ****P < 0.0001.

The antimetastatic efficacy of the combination therapy was assessed using a melanoma lung metastasis model via intravenous injection of B16F10 cells (Fig. 4A), reflecting the lung-predominant metastatic pattern of melanoma (*35*). Survival analyses highlighted the therapeutic benefit of the treatment regimens: Mice in the PBS group succumbed within 25 days, and Doxil moderately extended survival, whereas the Mn/MSA-2@Lipo + Doxil group achieved a complete survival rate beyond 40 days (Fig. 4B). Following treatment, lung metastatic nodules were counted. As shown in Fig. 4C, both the PBS and Mn/MSA-2@Lipo groups exhibited numerous metastatic nodules, whereas the Doxil group displayed a sparse number of nodules. Notably, the Mn/MSA-2@Lipo + Doxil group presented the fewest metastases, indicating a marked reduction in lung colonization. To further assess systemic disease burden, spleens and lymph nodes were excised and weighed. In the combination group, both spleen and lymph node weights were substantially lower than those in the PBS group, approaching levels observed in healthy controls (Fig. 4, D and E).

To prove the universality of this therapeutic strategy, we evaluated its efficacy in a 4T1 breast cancer model (Fig. 4F). Neither Mn/MSA-2@Lipo (25 mg/kg) nor Doxil (3 mg/kg) monotherapy effectively suppressed tumor growth. In contrast, their combination led to significant tumor shrinkage with the Mn^2+^-based formulation achieving nearly complete tumor regression after three treatment cycles (Fig. 4, G and H). Posttreatment monitoring revealed that the Mn/MSA-2@Lipo + Doxil group exhibited the slowest tumor recurrence. No significant body weight loss or histopathological abnormalities were observed (figs. S18 to S20), underscoring the safety and tolerability of this combination.

Collectively, these findings establish Mn/MSA-2@Lipo + Doxil as a potent and broadly applicable therapeutic strategy that enhances both local and systemic antitumor immunity, offering promise for effective treatment of primary tumors and metastatic disease across diverse cancer models.

### Coordinated DC maturation and antigen uptake maximizes combination therapy efficacy

DCs undergo extensive phenotypic and functional reprogramming upon maturation, marked by up-regulation of costimulatory molecules (e.g., CD80 and CD86) and down-regulation of antigen uptake capacity (*36*). Instead of acquiring additional antigens, mature DCs shift focus toward processing and cross-presenting previously internalized antigens to CD8^+^ T cells. To evaluate how this transition influences nanoadjuvant efficacy, we first examined the relationship between antigen uptake and DC maturation.

Fluorescence intensity increased steadily with concentrations from 20 to 200 μM during the first 12 hours and reached a plateau only when maturation markers began to appear (fig. S21). This plateau was not caused by saturation of the endocytic machinery as treatment with chlorpromazine reduced overall uptake but did not shift the plateau (fig. S22). These results suggest that, if DCs mature too early, before chemotherapy has released sufficient tumor antigens, their ability to prime CD8^+^ T cells may be compromised due to limited antigen internalization.

To test this hypothesis, we manipulated the interval between antigen exposure and nanoadjuvant-induced maturation in vitro.

**Fig. 5.**
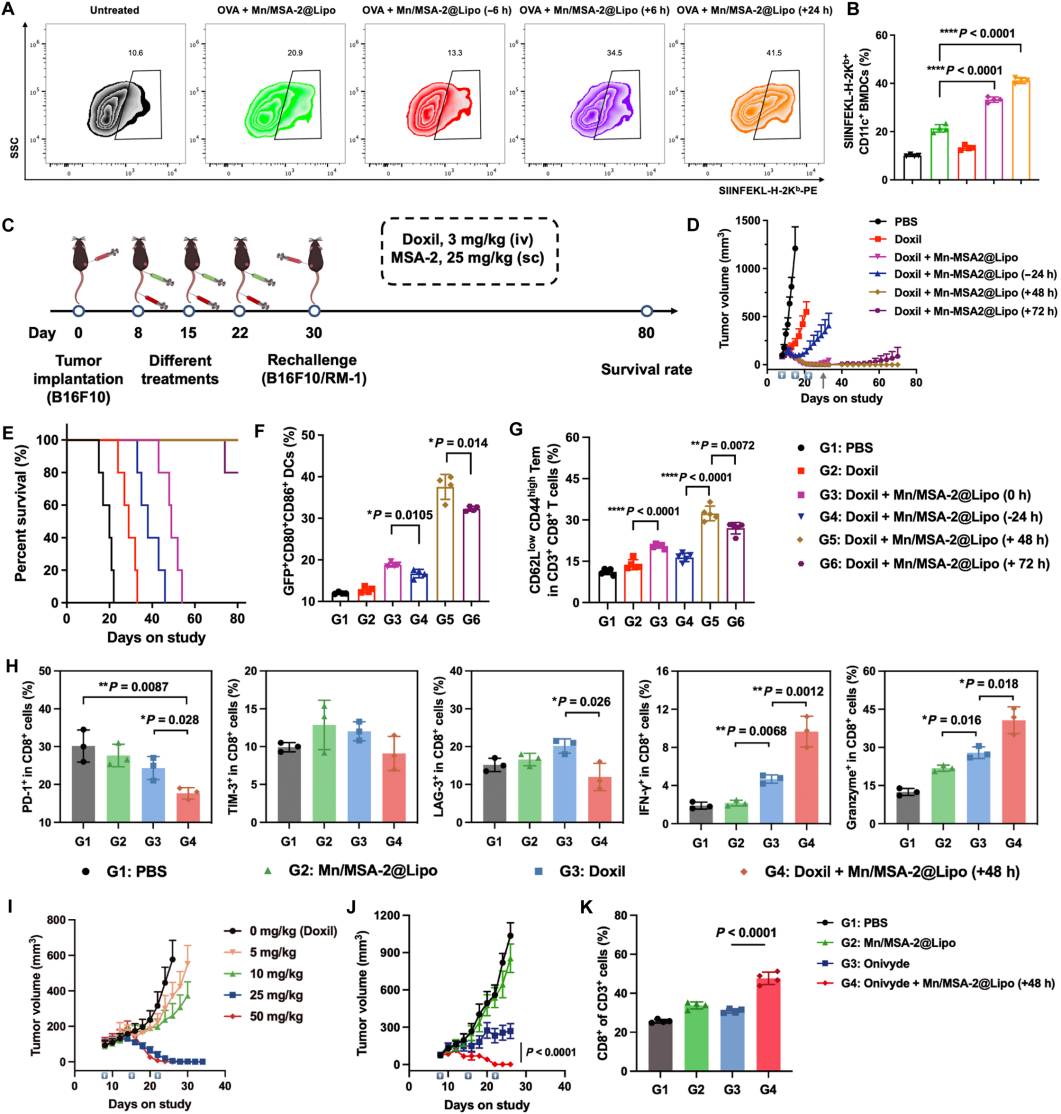
Temporally coordinated delivery of tumor antigens and adjuvants effectively activates antitumor immunity. (**A**) Representative flow cytometry scatterplots showing increased SIINFEKL-H-2K^b^ complex presentation by CD11c^+^ DCs following enhanced tumor antigen uptake. (**B**) Relative quantification of SIINFEKL-H-2K^b+^ CD11c^+^ DCs under different antigen internalization conditions. (**C**) Experimental design: B16F10 melanoma cells were subcutaneously inoculated into mice. On days 8, 15, and 22 postinoculation, tumor-bearing mice were treated with intravenous injections of Doxil (3 mg/kg) and subcutaneous injections of nanoadjuvants (25 mg/kg). On day 30, B16F10 melanoma or RM-1 prostate cancer cells were reinoculated on the opposite flank, and tumor growth was monitored. (**D**) Tumor growth curves of B16F10 melanoma in mice treated with different regimens. (**E**) Survival curves of mice under various treatment conditions. (**F**) Percentage of GFP^+^ mature DCs in tdLN following subcutaneous inoculation of B16F10-GFP cells and treatment with different regimens. (**G**) Relative quantification of CD3^+^CD8^+^CD62L^low^CD44^hi^ Tem in the spleen across different treatment groups. (**H**) Relative quantification of PD-1, TIM-3, LAG-3, IFN-γ, and granzyme B expression in tumor-infiltrating CD8^+^ T cells following the 48-hour chemoimmunotherapy regimen, as assessed by flow cytometry. (**I**) Tumor growth curves of B16F10 melanoma-bearing mice treated with Mn/MSA-2@Lipo at varying doses (5, 10, 25, or 50 mg/kg MSA-2 equivalent) administered 48 hours after Doxil. (**J**) Tumor growth curves of CT26 tumor-bearing mice receiving different treatments. (**K**) Flow cytometry analysis of the proportion of CD8^+^ T cells within the TME of CT26 tumor-bearing mice. Data are presented as means ± SD. Statistical significance was determined by one-way ANOVA with Tukey’s post hoc test. *P < 0.05, **P < 0.01, and ****P < 0.0001.

BMDCs were first pulsed with OVA and then stimulated with Mn/MSA-2@Lipo at either 0, 6, or 24 hours post-OVA loading. Delayed stimulation (6 and 24 hours) led to 1.6- and 1.9-fold increases, respectively, in SIINFEKL-H-2K^b^ presentation compared to simultaneous antigen and adjuvant exposure (Fig. 5, A and B). A parallel model using Doxil-treated, green fluorescent protein (GFP)–expressing B16F10 tumor cell debris confirmed that longer antigen uptake prior to maturation enhanced tumor antigen internalization and subsequent CD8^+^ T cell cytotoxicity (figs. S23 and S24). Maximal cytolytic activity occurred when DCs encountered debris 24 hours before Mn/MSA-2@Lipo stimulation.

To correlate these findings with in vivo antigen dynamics, we tracked the accumulation of GFP-tagged tumor antigen in tdLNs following intravenous Doxil administration in B16F10-GFP tumor-bearing mice. GFP fluorescence in tdLNs peaked at 48 hours postchemotherapy and declined thereafter (fig. S25), identifying this time point as the peak of antigen availability. We then tested different adjuvant administration schedules (24, 48, or 72 hours after Doxil) in B16F10-OVA mice and analyzed DC maturation and antigen presentation 24 hours later. Although all schedules increased CD80^+^CD86^+^ expression, only the 48-hour interval markedly enhanced SIINFEKL-H-2K^b^ presentation on CD11c^+^ DCs (fig. S26), establishing 48-hour postchemotherapy as the optimal priming window.

Collectively, these results underscore the importance of synchronizing DC maturation with peak antigen availability for effective combination immunotherapy. By administering nanoadjuvants at 48-hour postchemotherapy, we maximize antigen uptake, enhance cross-presentation, and improve CD8^+^ T cell activation, thereby substantially boosting overall therapeutic efficacy.

### Temporally programmed delivery maximizes chemotherapy and nanoadjuvant combination therapy

To determine how the timing of chemotherapy and nanoadjuvant delivery affects therapeutic efficacy, we systematically evaluated various administration intervals between Doxil and Mn/MSA-2@Lipo in the B16F10 melanoma model (Fig. 5C). When both agents were administered simultaneously, or when Mn/MSA-2@Lipo preceded Doxil, only marginal improvements in tumor suppression were observed relative to chemotherapy alone (Fig. 5, D and E, and fig. S27). These suboptimal outcomes likely result from premature DC maturation driven by early adjuvant administration, which reduces subsequent tumor antigen uptake and impairs CD8^+^ T cell priming. In contrast, sequential administration (delivering Mn/MSA-2@Lipo at either 48 or 72 hours after Doxil) substantially suppressed tumor growth, leading to complete tumor regression in all treated mice. Among these, the 48-hour interval exhibited superior efficacy, consistent with enhanced tumor antigen uptake by DCs and prolonged immune activation. To assess long-term immune protection, surviving mice were rechallenged with B16F10 tumor cells on day 30. Mice in the 48-hour treatment group displayed complete resistance to tumor regrowth, whereas those challenged with unrelated RM-1 prostate cancer cells developed tumors (fig. S28), demonstrating the specificity of the induced antitumor immune response.

To directly assess the phagocytic capacity of DCs following immunotherapy, we used a B16F10-GFP tumor model in which GFP fluorescence served as a surrogate for antigen uptake in tdLNs. Mice receiving Mn/MSA-2@Lipo 48 hours after Doxil injection yielded the highest proportion of GFP^+^CD80^+^CD86^+^ DCs in the tdLN, highlighting the importance of timing in maximizing DC-mediated antigen uptake and subsequent immune activation (Fig. 5F).

We next evaluated the durability of the antitumor immune response by examining splenic CD8^+^ effector memory T cells (Tem; CD3^+^CD8^+^CD62L^low^CD44^hi^) 40 days after completion of the optimized 48-hour chemoimmunotherapy regimen. Flow cytometry revealed a pronounced expansion of Tem in the 48-hour schedule group compared to both chemotherapy alone and untreated controls, demonstrating the induction of a long-lasting effector memory response (Fig. 5G and figs. S29 and S30). Flow cytometry analysis of tumor-infiltrating CD8^+^ T cells in the 48-hour regimen group revealed minimal PD-1 expression, indicating that these cells were not driven into exhaustion. At the same time, these CD8^+^ T cells exhibited strong production of IFN-γ and granzyme B, confirming a highly cytotoxic effector phenotype (Fig. 5H and fig. S31). These functional data demonstrate that the antitumor CD8^+^ T cells remain active and polyfunctional even after extended stimulation. Combined with the observed expansion of effector memory T cells, these findings reinforce that the optimized 48-hour regimen induces a durable, nonexhausted T cell response, underlining the long-term efficacy of this therapy.

To establish the optimal dosing of our nanoadjuvant, we performed a dose-titration study in the B16F10 melanoma model, administering Mn/MSA-2@Lipo at 5, 10, 25, or 50 mg/kg (MSA-2 equivalent) on the 48-hour schedule following Doxil. Only the 25 and 50 mg/kg doses led to substantial tumor regression, whereas lower doses were ineffective (Fig. 5I). Serum cytokine profiling further confirmed that doses between 25 and 50 mg/kg elicited strong IFN-β induction without triggering excessive IL-6 or TNF-α release, defining 25 to 50 mg/kg as the therapeutic window that maximizes efficacy while maintaining safety (fig. S32).

This temporally optimized strategy was also effective across multiple tumor models. In the CT26 colon carcinoma model, the same 48-hour schedule achieved durable tumor clearance (Fig. 5, J and K). Furthermore, replacing Doxil with paclitaxel or cisplatin while maintaining the 48-hour interval consistently yielded complete tumor eradication without recurrence (fig. S33).

These findings underscore that temporally programmed immunotherapy, in which nanoadjuvant delivery is scheduled to coincide with peak antigen availability 48 hours after chemotherapy, maximizes DC antigen uptake, strengthens the cross-talk between innate and adaptive immunity, and induces long-lasting, tumor-specific memory immunity across diverse therapeutic contexts.

## DISCUSSION

**Fig. 6.**
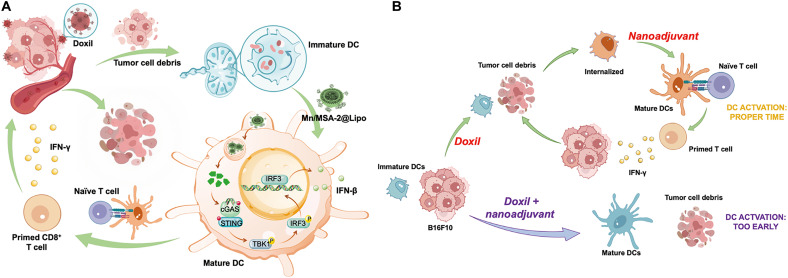
Schematic diagram. (**A**) Schematic of temporally programmed STING nanoadjuvant delivery unlocks synergistic chemotherapy-induced antitumor immunity. (**B**). Schematic representation of the temporally programmed delivery strategy. The top panel illustrates the optimal sequence of chemotherapy and nanoadjuvant delivery. Administering Mn/MSA-2@Lipo 48 hours after Doxil allows DCs to capture tumor antigens prior to maturation, enabling efficient cross-presentation to CD8^+^ T cells in the tdLNs. This strategy enhances CD8^+^ T cell activation, subsequent tumor cell destruction, and long-term immune memory. The bottom panel depicts premature adjuvant administration, leading to early DC maturation, reduced phagocytic capacity, and diminished antitumor immune responses.

The findings presented in this study demonstrate substantial progress in cancer immunotherapy through the integration of a lymph node–targeted STING agonist nanoadjuvant with a temporally programmed delivery strategy (Fig. 6A). To address the critical limitations of current STING agonist therapies, such as systemic toxicity and suboptimal antigen presentation, we designed Mn/MSA-2@Lipo, an 80-nm PEGylated lipid nanoadjuvant. This design leverages the principle that smaller nanoparticles (<100 nm) more readily traverse the pores of the extracellular matrix (ECM) after intradermal, subcutaneous, or intramuscular administration, leading to enhanced lymph node accumulation (*29*, *30*). In addition, PEGylation reduces nonspecific interactions with tissue components, further improving interstitial diffusion and lymphatic uptake, a strategy widely applicable to adjuvant delivery and immune modulation (*31*, *32*).

The development of Mn/MSA-2@Lipo as a nanoadjuvant highlights its potential to overcome delivery challenges inherent to STING agonists. Its high drug EE, stability, and precise lymph node targeting minimized systemic toxicity while enhancing localized immune responses. The inclusion of Mn^2+^ amplified STING activation by enhancing cyclic dinucleotide binding and downstream signaling. Although direct ICP-MS quantification of intracellular Mn^2+^ was not performed in this study, robust evidence of Mn^2+^-dependent STING activation was nonetheless observed. Elevated levels of phosphorylated STING, TBK1, and IRF3, along with increased IFN-β production, confirmed effective Mn^2+^-mediated amplification of the STING pathway. Future studies will include direct measurement of intracellular Mn^2+^ to further corroborate these findings. Furthermore, Mn/MSA-2@Lipo demonstrated a unique ability to enhance antigen presentation and stimulate T cell–mediated cytotoxicity, effectively bridging innate and adaptive immunity. This localized immune activation within lymph nodes underscores the importance of spatial targeting in improving therapeutic outcomes while reducing off-target effects.

The therapeutic efficacy of the combination therapy was validated in both localized and metastatic tumor models. Doxorubicin (DOX) hydrochloride, an anthracycline antibiotic, is one of the most effective antineoplastic agents used as a “first-line” drug in various types of malignancies, including ovarian and metastatic breast cancer (*37*, *38*); in our studies, it was delivered as liposomal Doxil in the breast cancer model. In poorly immunogenic melanoma and breast cancer models, the combined treatment achieved complete tumor regression in most treated mice and provided durable protection against tumor recurrence. In addition, this approach effectively reduced pulmonary metastases in a melanoma model, addressing a key challenge in clinical oncology. These benefits were achieved without notable weight loss or systemic toxicity, highlighting the safety profile of the approach. The improved survival outcomes and tumor-specific immune memory elicited by the therapy highlight its potential to address tumor heterogeneity and overcome immune resistance.

A central insight from this work is the importance of temporal coordination between chemotherapy and immunotherapy. Administering chemotherapy prior to nanoadjuvant delivery, at a carefully defined interval, allowed tumor cell death to generate and release antigens, which were subsequently captured by DCs during their optimal functional window. Specifically, delivering Mn/MSA-2@Lipo 48 hours after chemotherapy maximized antigen uptake and cross-presentation by DCs, thereby enhancing CD8^+^ T cell activation. In contrast, simultaneous administration or reverse sequencing diminished efficacy, likely due to premature DC maturation that impaired antigen acquisition. These findings highlight the importance of aligning immune activation with peak antigen availability to optimize immunotherapy outcomes.

Mechanistically, the study revealed that the enhanced efficacy of the combination therapy stemmed from the precise coordination of DC maturation and antigen uptake. Mature DCs lose their capacity for antigen capture, highlighting the necessity of aligning immune activation with peak antigen availability (Fig. 6B). Temporal coordination not only improved antigen cross-presentation but also strengthened cytotoxic T cell response, as evidenced by increased CD8^+^ T cell infiltration and elevated CD8^+^/CD4^+^ T cell ratios within tumor tissues. These findings suggest that modulating the timing of immune stimulation can effectively counteract immune suppression within the TME, a major barrier to successful immunotherapy.

In conclusion, the development and optimization of Mn/MSA-2@Lipo within a temporally programmed therapeutic regimen addresses long-standing barriers in STING-based immunotherapy. By enhancing spatial targeting to lymph nodes and synchronizing immune activation with peak tumor antigen release, this approach substantially improves treatment efficacy while maintaining a favorable safety profile. These findings offer a robust and generalizable strategy for integrating nanotechnology with immunotherapy and underscore the importance of both spatial and temporal precision in designing effective combination treatments. The results also provide a foundation for broader application across tumor types and chemotherapeutic agents, supporting continued efforts toward clinical translation.

## MATERIALS AND METHODS

### Materials

Hydrogenated soybean phospholipids (HSPC), cholesterol, and 2-distearoyl-*sn*-glycero-3-phosphoethanolamine-*N*-[methoxy(polyethylene glycol)-2000] (DSPE-mPEG_2000_) were purchased from AVT (Shanghai) Pharmaceutical Tech Co. Ltd. Sepharose CL-4B gel and the lactate dehydrogenase (LDH; #BC0685) activity assay kit were obtained from Beijing Solarbio Science & Technology Co. Ltd. Dulbecco’s modified Eagle’s medium (DMEM), RPMI 1640, and fetal bovine serum (FBS) were sourced from Dalian Meilun Biotechnology Co. Ltd. Recombinant granulocyte-macrophage colony-stimulating factor (GM-CSF) and IL-4 were purchased from Elabscience Biotechnology Co. Ltd. Cell culture dishes and plates were supplied by Wuxi NEST Biotechnology Co. Ltd. The BCA protein assay kit was obtained from Beyotime Biotechnology Co. Ltd. (Shanghai, China), and enzyme-linked immunosorbent assay (ELISA) kits for IFN-β, TNF-α, and IL-6 were acquired from Neobioscience Technology Co. Ltd. β-Actin (D6A8, 1:1000), STING (D2P2F), phospho-STING (Ser^366^, E9A9K), TBK1 (D1B4), phospho-TBK1 (Ser^172^, D52C2), IRF3 (D6I4C), phospho-IRF3 (S396, D6O1M), and goat anti-rabbit horseradish peroxidase (HRP) secondary antibody were purchased from Cell Signaling Technology (MA, USA). FVS-AF700 (catalog no. 564997, RRID: AB_2869637), CD16/CD32 (catalog no. 553141, RRID: AB_394656), APC-Cy7-CD45 (catalog no. 557659, RRID: AB_396774), PE-CD80 (catalog no. 553769, RRID: AB_395039), APC-CD80 (catalog no. 560016, RRID: AB_1645212), PE-CD86 (catalog no. 551396, RRID: AB_394180), APC-CD86 (catalog no. 558703, RRID: AB_2075114), PE-SIINFEKL-H-2K^b^ (catalog no. 569792, RRID: AB_3685327), PE-CD4 (catalog no. 557308, RRID: AB_396634), APC-CD8 (catalog no. 569870, RRID: AB_3685380), PE-CD8 (catalog no. 568906, RRID: AB_3684622), PerCP-Cy5.5-CD8 (catalog no. 551162, RRID: AB_394081), APC-CD44 (catalog no. 559250, RRID: AB_398661), PE-CD62L (catalog no. 561918, RRID: AB_394666), BV480–IFN-γ (catalog no. 566097, RRID: AB_2739549), BV650-CD223 (LAG-3, catalog no. 740560, RRID: AB_2740261), PerCP-Cy5.5-CD279 (PD-1, catalog no. 561273, RRID: AB_10645786), and BV421-CD366 (TIM-3, catalog no. 747626, RRID: AB_2744192) antibodies were purchased from BD Bioscience (CA, USA). BV650-MHC II (catalog no. 107641, RRID: AB_2565975), FITC-CD3 (catalog no. 100203, RRID: AB_312660), and PerCP-Cy5.5-CD3 (catalog no. 100218, RRID: AB_1595492) antibodies were purchased from BioLegend Inc. (CA, USA).

### Cell lines and cell culture

The human monocyte cell line THP-1 (catalog no. CL-0233) was purchased from Procell Life Science & Technology Co. Ltd. The mouse melanoma cell line B16F10 (CSTR:19375.09.3101MOUTCM36) and the mouse breast cancer cell line 4T1 (CSTR:19375.09.3101MOUTCM32) were obtained from the Cell Bank of the Chinese Academy of Sciences (Shanghai, China). The B16F10-GFP cell line (catalog no. TCM-C713LG) was purchased from Haixing Biosciences Co. Ltd. (Suzhou, China). These cells were cultured in RPMI 1640 medium supplemented with 10% FBS, penicillin (100 U/ml), and streptomycin (100 μg/ml). Primary BMDCs were isolated from male C57BL/6 mice. Bone marrow was harvested by flushing the femurs with cold PBS, and cells were cultured in petri dishes with DMEM containing 10% FBS, penicillin (100 U/ml), streptomycin (100 μg/ml), IL-4 (10 ng/ml), and GM-CSF (20 ng/ml). The medium was partially refreshed on day 2 and fully replaced on day 4. Nonadherent cells, which comprised over 75% CD11c^+^ cells as determined by flow cytometry, were collected for experiments on day 6. All cells were maintained at 37°C in a humidified atmosphere with 5% CO_2_.

### Ethical statement

All experimental procedures were conducted in compliance with the ethical guidelines approved by the Institutional Animal Care and Use Committee of Shenyang Pharmaceutical University (SYPU-IACUC-S2023-1213-102).

### Animal studies

C57BL/6 (male, 4 to 6 weeks, 20 to 22 g) and Balb/c (female, 4 to 6 weeks, 18 to 20 g) mice were purchased from Changsheng Biotechnology (Shenyang, China). Mice were maintained in a specific pathogen–free facility under controlled temperature (23° to 26°C), humidity (40 to 60%), and a 12-hour/12-hour light/dark cycle, with ad libitum access to food and water. At the study endpoint, animals were euthanized by CO_2_ inhalation, followed by cervical dislocation.

### Preparation and characterization of nanoliposomes

MSA-2 liposomes were prepared using the ethanol injection method (*39*). A lipid mixture containing HSPC, cholesterol, and DSPE-mPEG_2000_ at a mass ratio of 3:1:0.05 was dissolved in ethanol and rapidly injected into an aqueous solution containing metal ions, yielding stable liposomes at a total lipid concentration of 10 mg/ml. After dialysis to replace the aqueous phase, liposomes were incubated with MSA-2 at 60°C for 30 min, cooled in an ice bath for 15 min, and stored under nitrogen at 4°C. The concentration of MSA-2 was determined by high-performance liquid chromatography (HPLC). Particle size, PDI, and zeta potential of Mn/MSA-2@Lipo were analyzed using dynamic light scattering on a Malvern Zetasizer, with data processed in Zetasizer software (version 7.13). EE was determined by HPLC after removing unencapsulated MSA-2 via Sepharose CL-4B gel column chromatography. The EE was calculated as follows

$$\begin{array}{c} \text{EE}\;\left(\%\right)=W_{\text{MSA-}2\;\text{in}\;\text{Mn}/\text{MSA-}2@\text{Lipo}}/W_{\text{initial}\;\text{MSA-}2\;\text{added}}\times 100 \end{array}$$

where *W*_MSA-2 in Mn/MSA-2@Lipo_ and *W*_initial MSA-2 added_ represent the weight of encapsulated MSA-2 and the initially added MSA-2, respectively. TEM images were acquired by applying Mn/MSA-2@Lipo onto a carbon-coated cooper grid and visualized using a JEOL 100CX II TEM at 200 kV. For cryo-TEM, diluted Mn/MSA-2@Lipo was flash-frozen in a liquid ethane-methane mixture and observed with an FEI Talos F200C electron microscope at 200 kV. Mn/MSA-2@Lipo was sterilized via filtration and stored under nitrogen at 4°C for up to 30 days. Stability was assessed by measuring particle size and EE at scheduled intervals.

### In vitro cellular uptake and activation of BMDCs

BMDCs were seeded in a 12-well plate (4 × 10^5^ cells per well) and treated with different formulations at an MSA-2 concentration of 40 μM. Cellular uptake was evaluated by flow cytometry using a 405-nm excitation laser and a 450/40-nm filter.

To assess the maturation status of DCs following treatment, BMDCs were seeded in 12-well plates (4 × 10^5^ cells per well) and treated with either nanoliposomes or free MSA-2 at an MSA-2 concentration of 40 μM. Following 6 hours of incubation, cells were collected, washed twice with PBS, and labeled with fluorescent antibodies: APC-Cy7-CD45, FITC-CD11c, PE-CD80, APC-CD86, and BV650-MHC II. Stained cells were analyzed via flow cytometry (FACSCalibur, BD Biosciences, San Jose, CA).

### Cross-presentation of antigens by BMDCs

BMDCs were treated with either free MSA-2 or nanoliposomes for 6 hours at an MSA-2 concentration of 40 μM, followed by overnight incubation with 40 nM OVA_257-264_ peptide (SIINFEKL). The cells were then labeled with a phycoerythrin (PE)–conjugated 25-D1.16 monoclonal antibody specific for SIINFEKL-H-2K^b^ complexes. The mean fluorescence intensity (MFI) of PE was measured in DCs by flow cytometry to assess antigen cross-presentation (FACSCalibur, BD Biosciences, San Jose, CA).

### Cytolytic analysis of splenocytes primed by BMDCs

To assess the cytolytic capacity of CD8^+^ T cells activated by BMDCs, B16F10 cells were used as target cells. B16F10 cells were seeded into 48-well plates at a density of 1 × 10^4^ cells per well. BMDCs were treated with different formulations for 6 hours at an MSA-2 concentration of 40 μM, followed by an overnight incubation with 40 nM OVA_257-264_ peptide (SIINFEKL). Splenocytes from C57BL/6 mice were prepared as single-cell suspensions and cocultured with BMDCs at a 2:1 ratio (BMDCs = 5 × 10^4^ cells) in the presence of B16F10 cells. A coculture of BMDCs and B16F10 cells was used as a negative control. After 48 hours of incubation, the supernatant was collected, centrifuged to remove the debris, and analyzed with an LDH cytotoxicity assay kit.

### Western blot analysis

To evaluate the expression and phosphorylation levels of TBK1, IRF3, and STING, human THP1 cells were seeded in 6-well plates at a density of 2 × 10^6^ cells per well and treated with either free MSA-2 or nanoliposomes at a concentration of 40 μM for 6 hours. Cells were lysed using radioimmunoprecipitation assay buffer, and protein concentrations were determined by BCA assay. Proteins were separated via SDS–polyacrylamide gel electrophoresis, transferred onto polyvinylidene difluoride membranes, and incubated overnight at 4°C with primary antibodies targeting TBK1, phospho-TBK1, IRF3, phospho-IRF3, STING, and phospho-STING. After washing, membranes were incubated with goat anti-rabbit HRP–conjugated secondary antibody and visualized using an ECL substrate on a chemiluminescence imaging system.

### In vivo antitumor efficacy

B16F10 tumor-bearing mice were subcutaneously inoculated with 5 × 10^5^ cells in the right flank. Upon reaching a tumor volume of 60 to 80 mm^3^, mice were randomized into five groups (*n* = 10 per group). Treatment involved three injections of intravenous Doxil (3 mg/kg) and subcutaneous nanoadjuvant (25 mg/kg) on days 8, 15, and 22. At the study’s endpoint, mice were euthanized, and tumors as well as tdLNs were collected. For flow cytometry analysis, tumors and tdLNs were dissociated, digested with deoxyribonuclease I and collagenase IV, and filtered through a 70-μm strainer to obtain single-cell suspensions. Cells were stained with anti-CD45, anti-CD3, anti-CD4, anti-CD8 anti-CD44, anti-CD62L, anti–IFN-γ, anti–LAG-3, anti–PD-1, and anti–TIM-3 antibodies to assess T cell infiltration. DC maturation was analyzed using anti-CD11c, anti-CD80, and anti-CD86 antibodies.

### Study of temporally programmed delivery of chemotherapy and nanoadjuvant

C57BL/6 mice bearing subcutaneous B16F10 tumors were randomized into six groups once tumor reached 60 to 80 mm^3^ (*n* = 10 per group). On days 8, 15, and 22 after tumor implantation, mice received intravenous injections of Doxil (3 mg/kg), followed by subcutaneous administration of Mn/MSA-2@Lipo either 24 hours before, at the same times as, or 48 or 72 hours after Doxil treatment. The Doxil-only group served as the control. Tumor growth and mouse survival were monitored posttreatment. To assess the long-term immune memory effects of the temporally programmed delivery strategy, cured B16F10 tumor-bearing mice from the Doxil + Mn/MSA-2@Lipo (+48 hours) group were rechallenged with either B16F10 or 4T1 cells subcutaneously on day 30 following complete tumor regression.

### Systemic toxicity of Mn/MSA-2@Lipo

Biosafety evaluations were conducted in tumor-bearing C57BL/6 and Balb/c mice. Throughout the treatment period, body weight and tumor volume were monitored. At the study’s conclusion, serum was collected for hepatorenal function tests, and major organs (heart, liver, spleen, lung, and kidney) were harvested for histological analysis with H&E staining. Untreated mice served as the control group.

### Statistical analysis

Multiple group comparisons were performed using one-way analysis of variance (ANOVA) followed by Tukey’s multiple-comparisons test. Survival analyses were conducted using the log-rank test. All results are presented as means ± SD, with *P* values and significance denoted in the figures [not significant (n.s.) > 0.05, *P < 0.05, **P < 0.01, ***P < 0.001, and ****P < 0.0001].
