# Supplementary material for: Temporally programmed STING nanoadjuvant delivery unlocks synergistic chemotherapy-induced antitumor immunity
Source: Sci Adv. 2025 Jul 18;11(29):eadw0797. doi: 10.1126/sciadv.adw0797 (PMC12273768; doi:10.1126/sciadv.adw0797)
Supplement: Supplementary file 1 — Figs. S1 to S33 [file sciadv.adw0797_sm.pdf]

Science Advances
AAAS

Supplementary Materials for

Temporally programmed STING nanoadjuvant delivery unlocks synergistic chemotherapy-induced antitumor immunity

Zimeng Yang *et al.*

Corresponding author: Yongjun Wang, wangyongjun@syphu.edu.cn; Zhonggui He, hezhonggui@syphu.edu.cn; Hongzhuo Liu, liuhongzhuo@syphu.edu.cn

Sci. Adv. **11**, eadw0797 (2025)
DOI: 10.1126/sciadv.adw0797

This PDF file includes:

Figs. S1 to S33

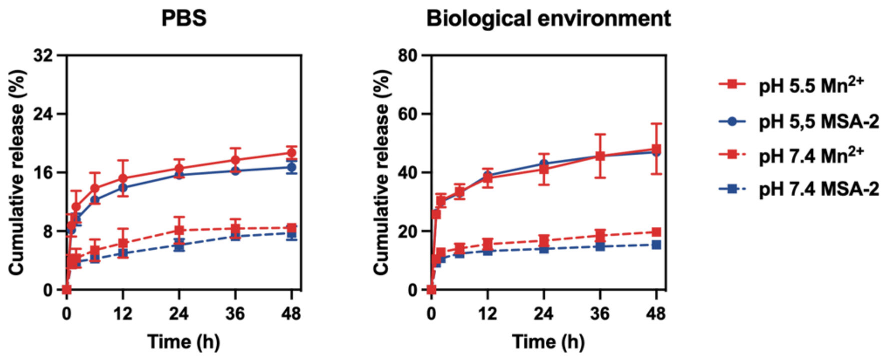

Supplementary Fig. 1. Sustained release of Mn/MSA-2@Lipo under physiological conditions. Cumulative release of Mn/MSA-2@Lipo was measured in PBS and a biological environment (n = 3).

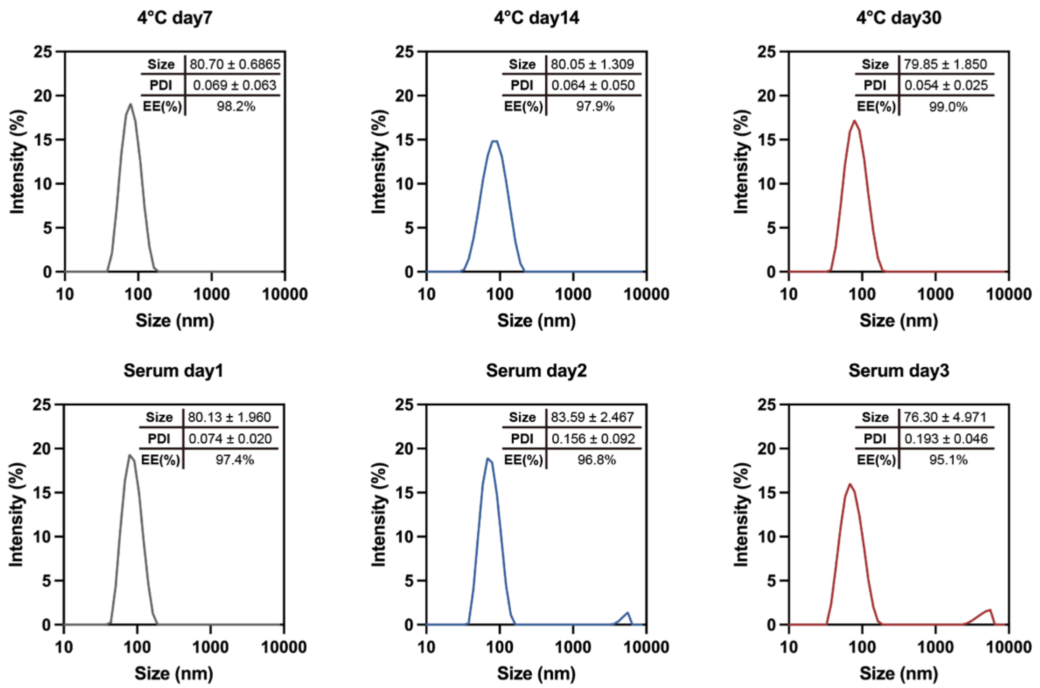

Supplementary Fig. 2. Stability of Mn/MSA-2@Lipo under storage and physiological conditions. Liposome stability was assessed under standard storage and physiological environments (n = 3).

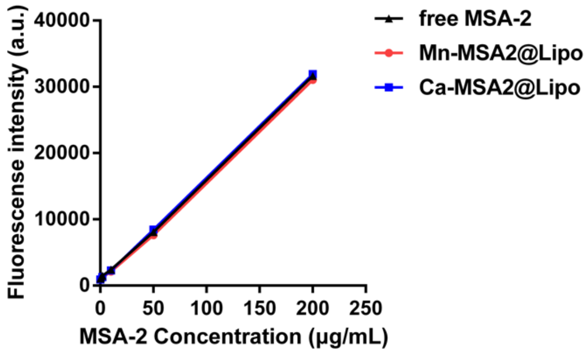

Supplementary Fig. 3. Fluorescence intensity profiles of MSA-2 and nanoliposomes in methanol. Fluorescence intensities were compared across concentrations for different formulations (Ex = 405 nm, Em = 450 nm, n = 3).

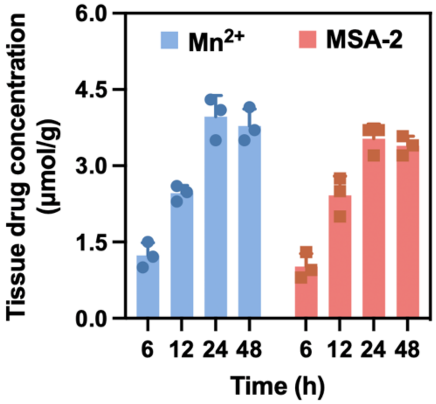

Supplementary Fig. 4. Quantification of Mn^{2+} and MSA-2 levels in draining lymph nodes. Mn^{2+} and MSA-2 concentrations were measured at various time points post-injection using ICP-MS and LC-MS, respectively (n = 3).

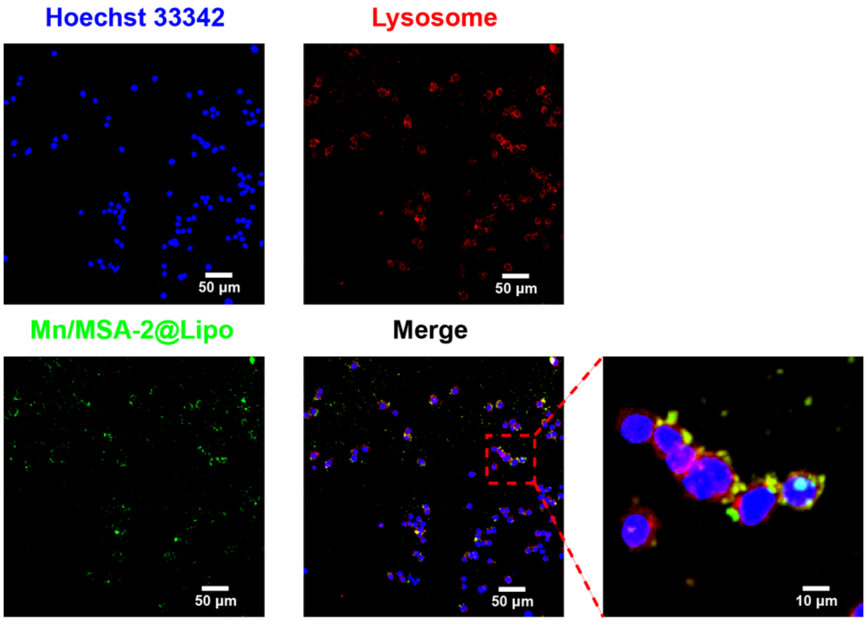

Supplementary Fig. 5. Confocal microscopy reveals lysosomal escape of Mn/MSA-2@Lipo in BMDCs. Nuclei are stained in blue, nanoadjuvant in green, and lysosomes in red. Nanoadjuvant localization in BMDCs demonstrates endosomal escape for cytosolic delivery.

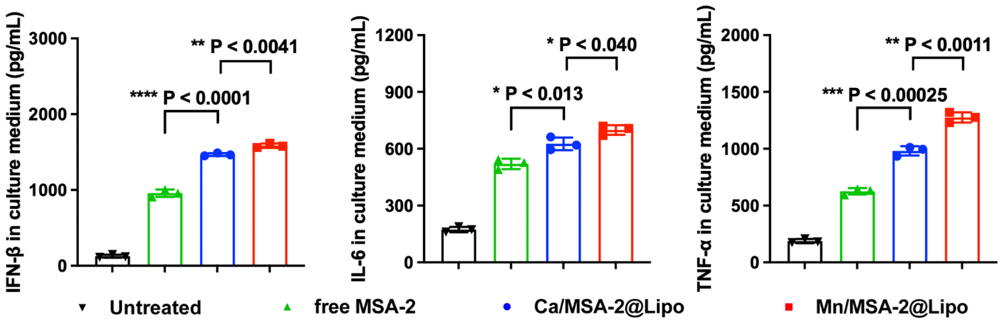

Supplementary Fig. 6. MSA-2 induces proinflammatory cytokine secretion in THP-1 cells. Levels of IFN-β, IL-6 and TNF-α in THP-1 culture supernatants following treatment with 40 μM MSA-2 (n = 3).

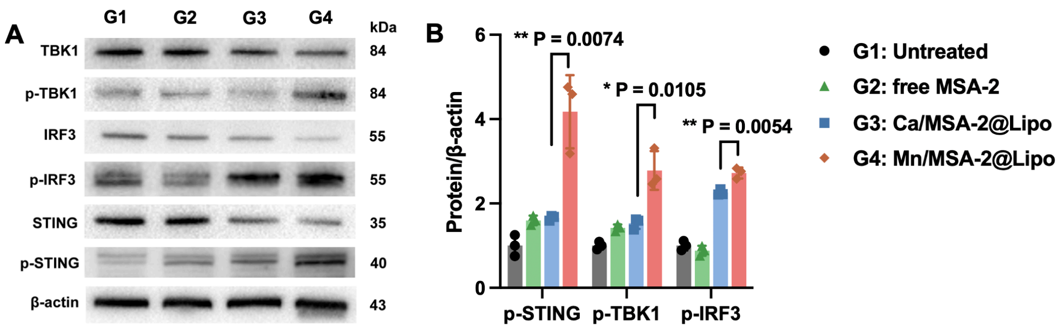

Supplementary Fig. 7. MSA-2 nanoliposomes activate the STING pathway in THP-1 cells. (**A**) Western blot analysis of STING pathway activation in THP1 cells treated with free MSA-2 or MSA-2-loaded nanoliposomes for 6 hours. (**B**) Quantification of protein expression levels from (A). Data are representative of three independent experiments.

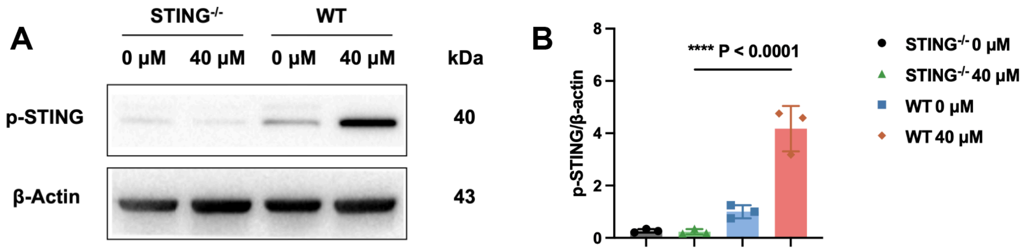

Supplementary Fig. 8. Mn/MSA-2@Lipo does not activate STING signaling in $STING^{-/-}$ cells. (**A**) Western blot analysis of p-STING expression in STING-knockout ($STING^{-/-}$) THP-1 cells following incubation with Mn/MSA-2@Lipo. (**B**) Quantification of p-STING protein levels from (A). Data are representative of three independent experiments.

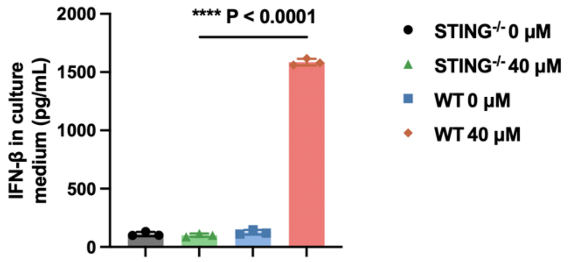

Supplementary Fig. 9. Lack of IFN-β production in STING$^{-/-}$ cells after Mn/MSA-2@Lipo treatment. Concentration of IFN-β in the culture medium of STING-knockout THP-1 cells following incubation with the nanoadjuvant (n = 3).

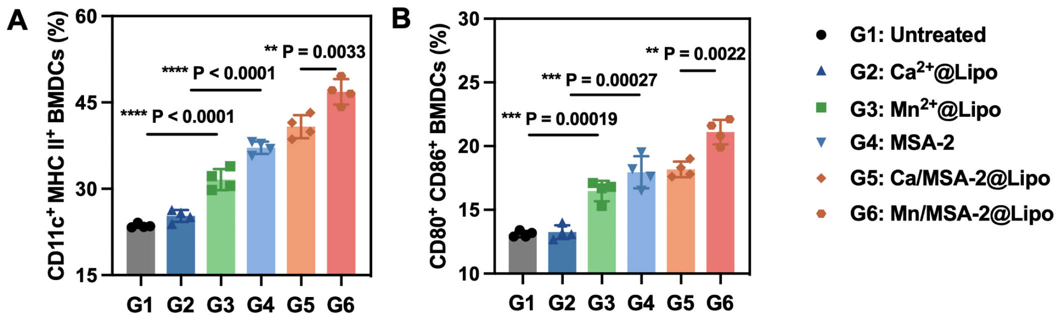

Supplementary Fig. 10. Mn/MSA-2@Lipo promotes DC maturation in tumor-free mice. Proportion of MHC II^+ (**A**) and $CD80^+CD86^+$ (**B**) DCs cells in the tumor-draining lymph nodes (tdLN) of tumor-free mice after subcutaneous administration of Mn/MSA-2@Lipo ($n = 4$).

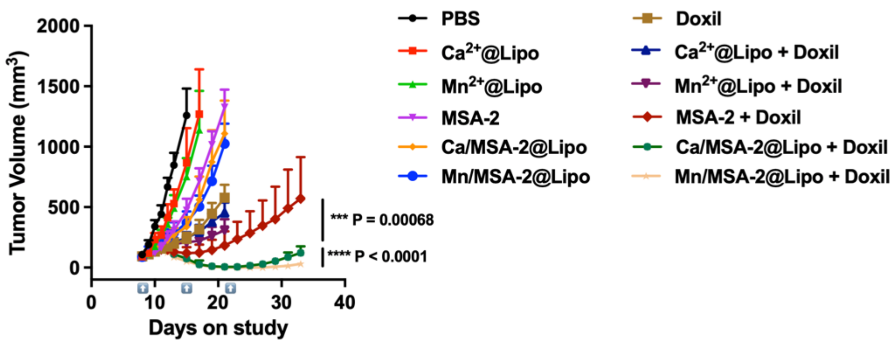

Supplementary Fig. 11. Combination therapy suppresses tumor growth in B16F10 melanoma model. Tumor growth curves of B16F10 tumor-bearing mice receiving different treatments. Data are represented as mean ± SD (n = 10).

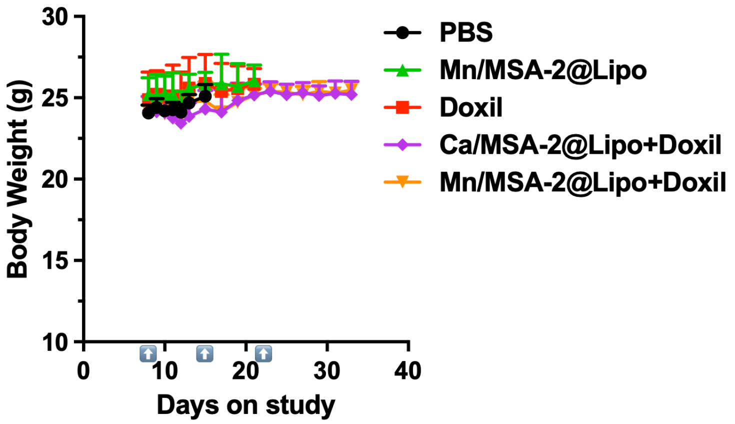

Supplementary Fig. 12. Body weight profiles remain stable following treatment in melanoma model. Weights of mice receiving various treatments were monitored (n = 10).

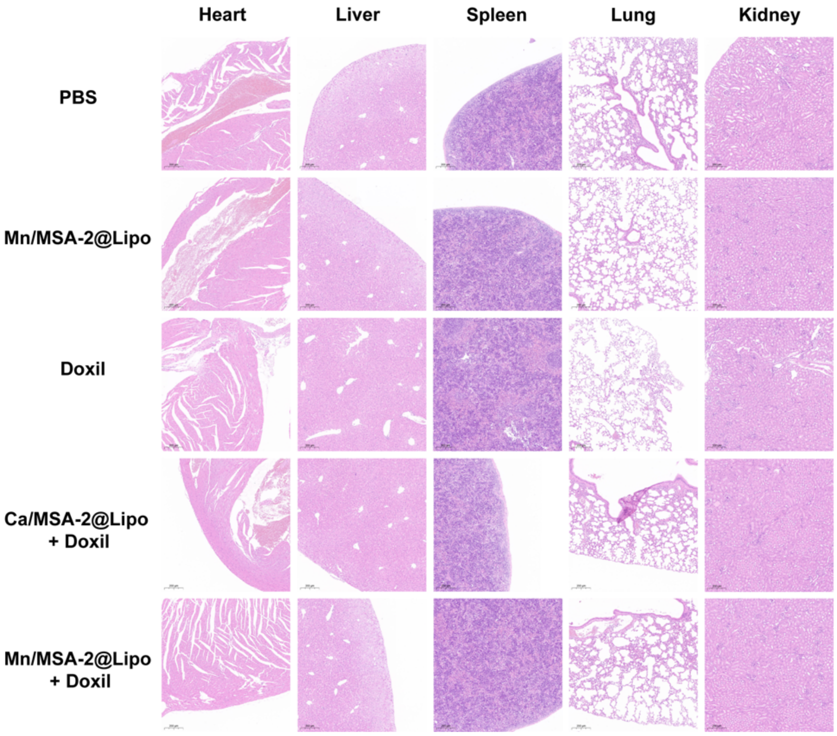

Supplementary Fig. 13. Histological analysis of major organs confirms biosafety of treatment. H&E staining was performed on organ sections after treatment.

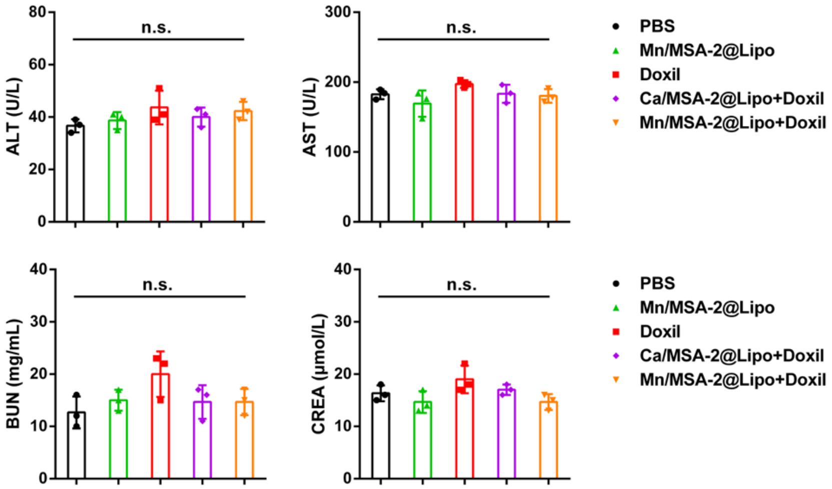

Supplementary Fig. 14. Liver and kidney function remain within normal ranges post-treatment. ALT, AST, BUN, and CREA levels were assessed (n = 3).

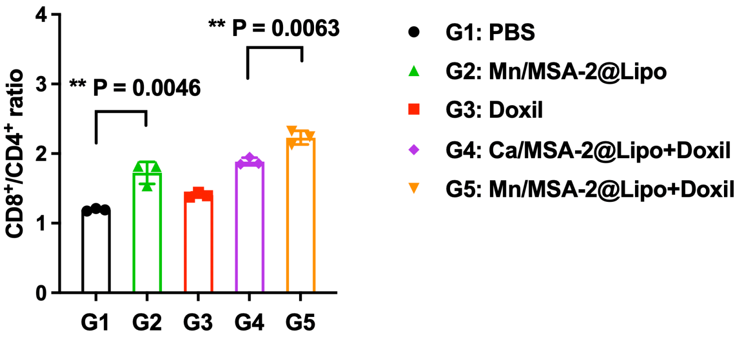

Supplementary Fig. 15. Combination therapy increases $CD8^+/CD4^+$ T cell ratio in tumor. Flow cytometry was used to assess T cell subset ratios (n = 3).

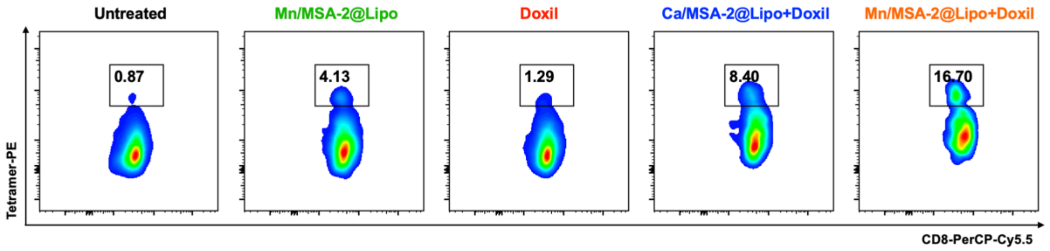

Supplementary Fig. 16. Representative flow cytometry of antigen-specific CD8$^+$ T cells in blood. SIINFEKL-H-2K^b tetramer$^+$ CD8$^+$ T cells were visualized in peripheral blood.

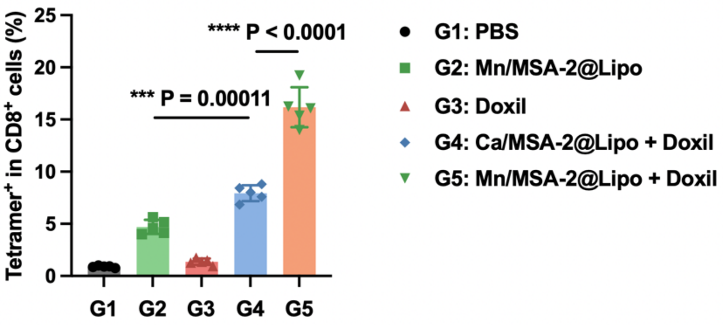

Supplementary Fig. 17. Quantification of antigen-specific $CD8^+$ T cells post-treatment. $Tetramer^+$ $CD8^+$ T cells were expressed as a percentage of total $CD8^+$ cells (n = 3).

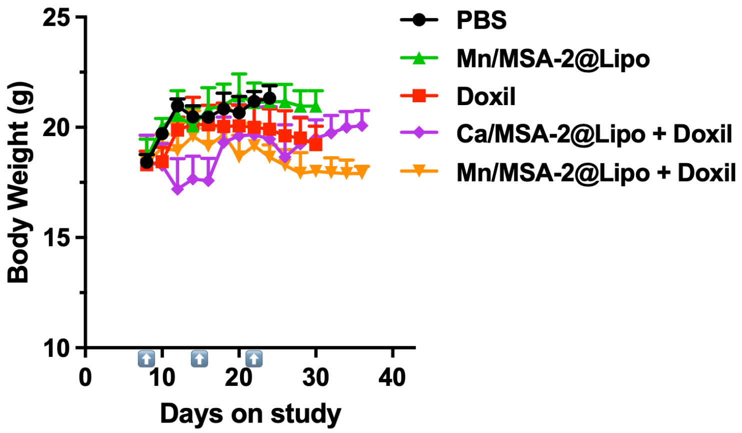

Supplementary Fig. 18. Stable body weights observed in 4T1 breast cancer model. Mice tolerated treatment well without significant weight loss (n = 5).

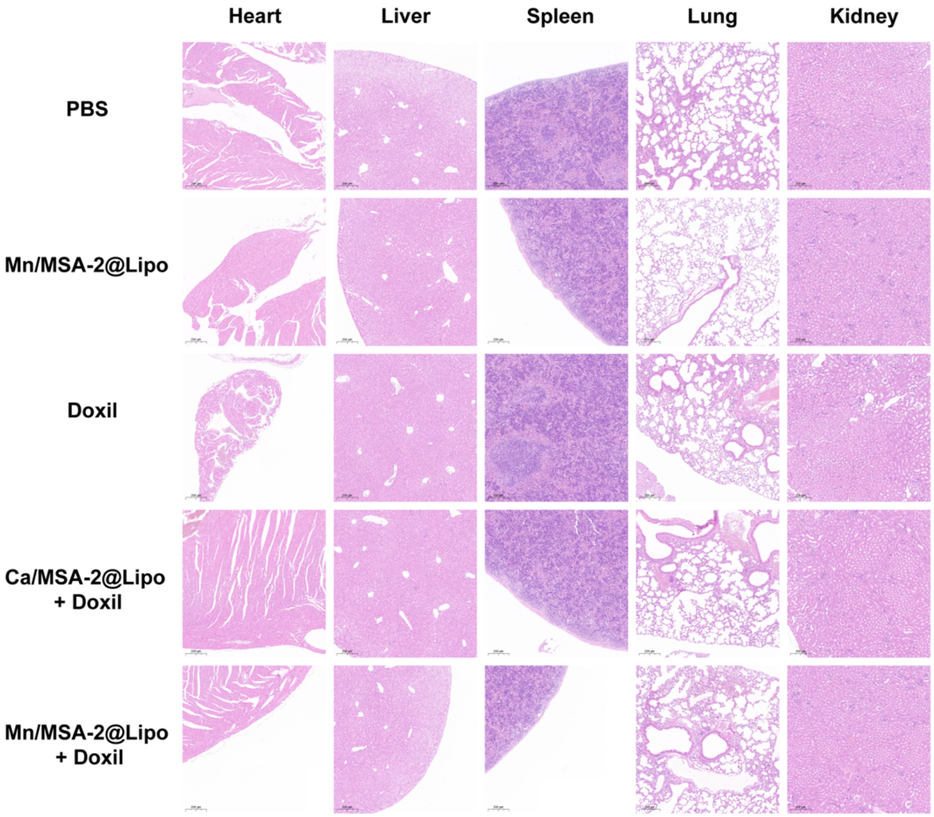

Supplementary Fig. 19. Histological evaluation of organs confirms safety in breast cancer model. H&E staining was performed on treated mice from the 4T1 model.

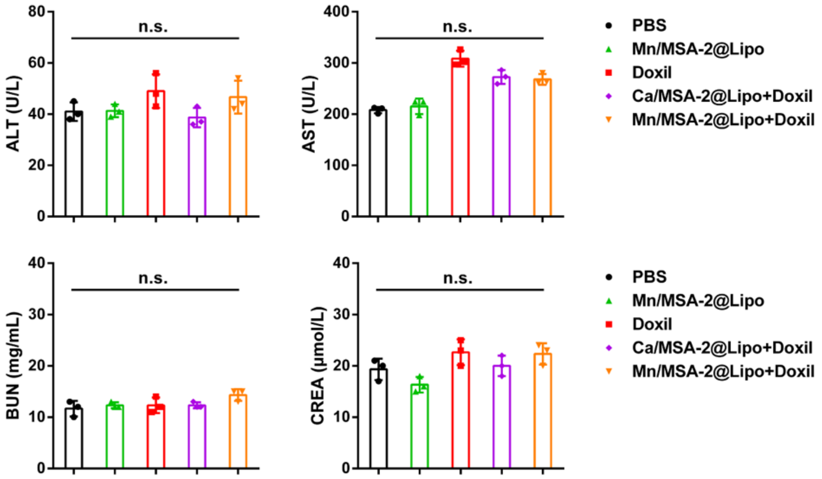

Supplementary Fig. 20. Hepatorenal parameters remain within safe limits in breast cancer model. ALT, AST, BUN, and CREA levels were measured (n = 3).

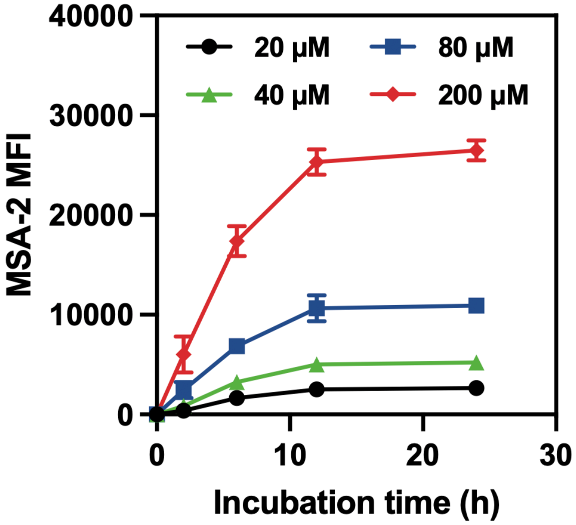

Supplementary Fig. 21. DC uptake of Mn/MSA-2@Lipo increases with dose. Mean fluorescence intensity of BMDCs after incubation with varying concentrations of Mn/MSA-2@Lipo for 24 hours. Data are represented as mean ± SD (n = 3).

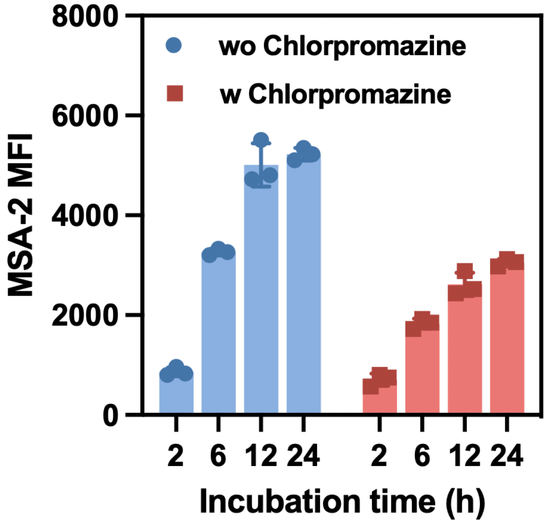

Supplementary Fig. 22. Endocytosis inhibition reduces nanoadjuvant uptake in BMDCs. Chlorpromazine pretreatment suppressed uptake efficiency (n = 3).

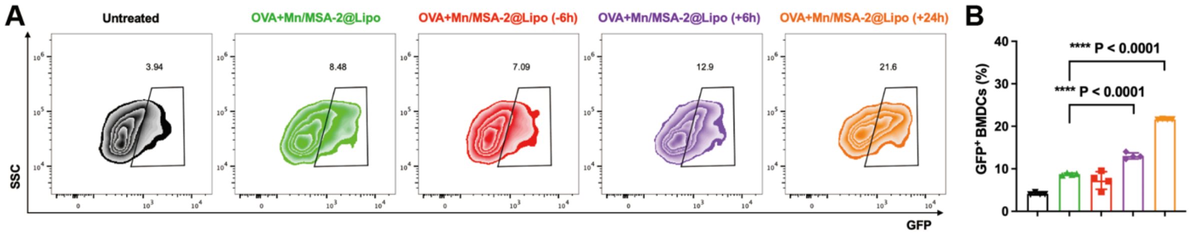

Supplementary Fig. 23. Increased internalization of tumor antigens enhances the ability of mature dendritic cells to present tumor-derived debris (GFP). (**A**) Representative flow cytometry scatter plots showing GFP signal in mature DCs. (**B**) Quantification of GFP^+ DCs, indicating enhanced antigen presentation following increased antigen uptake. Data are represented as mean ± SD (n = 4).

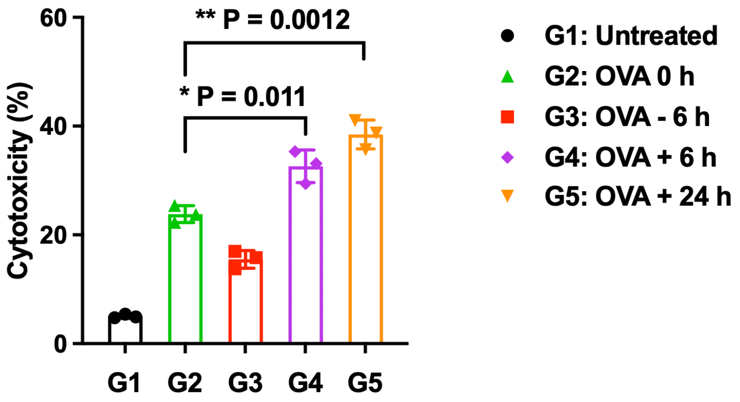

Supplementary Fig. 24. T cell cytotoxicity depends on antigen and nanoadjuvant timing. The cytolytic activity of T cells against B16F10 cells after OVA and Mn/MSA-2@Lipo were added to BMDCs at different intervals. Data are represented as mean ± SD (n = 3).

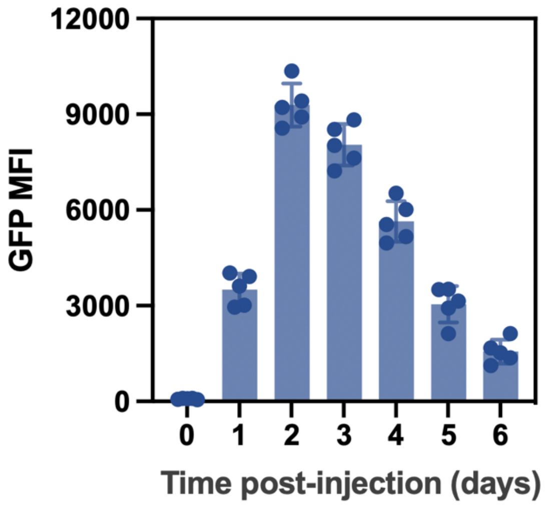

Supplementary Fig. 25. Antigen presence in tdLNs peaks after 48 h of Doxil administration. GFP fluorescence was measured to assess antigen accumulation (n = 5).

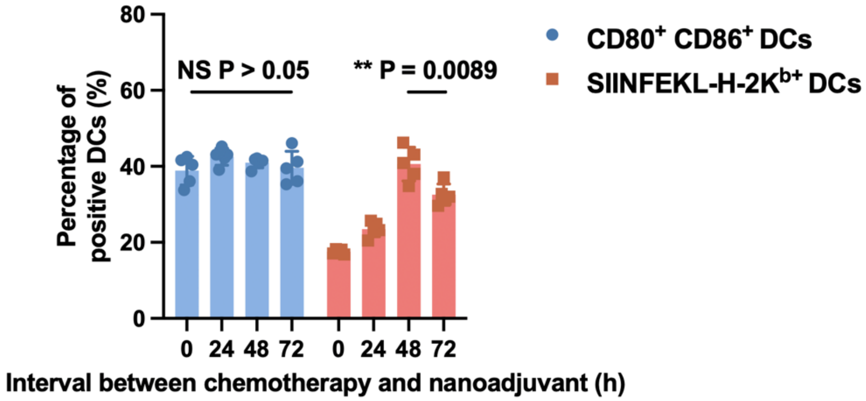

Supplementary Fig. 26. DC maturation and antigen presentation depend on treatment interval. Flow cytometry analysis of tdLNs from B16F10-OVA tumor-bearing mice. Mice received intravenous Doxil followed by subcutaneous injection of Mn/MSA-2@Lipo at 0, 24, 48, or 72 hours. Twenty-four hours after nanoadjuvant administration, tdLNs were collected and assessed for DCs maturation markers (CD80, CD86) and antigen presentation (SIINFEKL-H-2K^b complex).

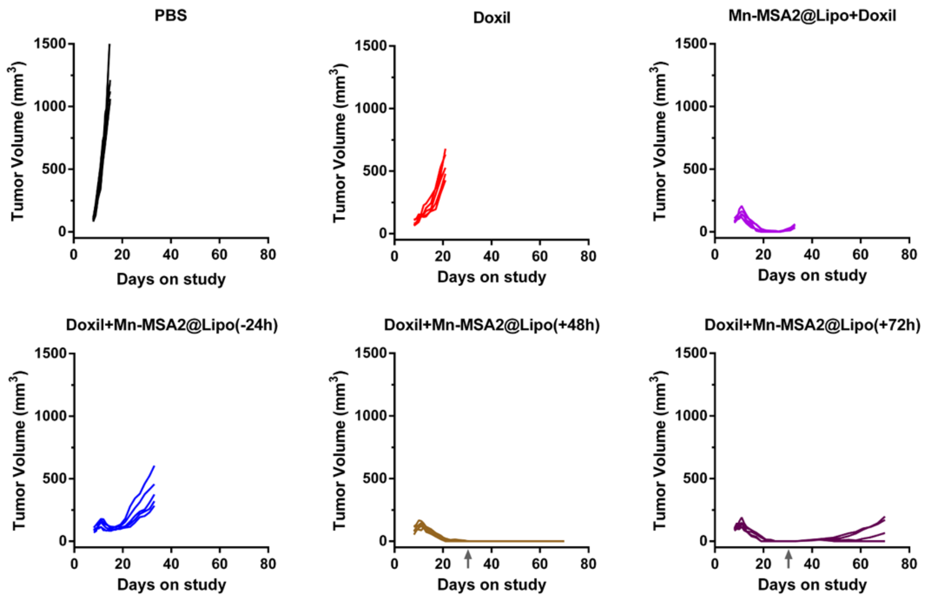

Supplementary Fig. 27. Individual tumor growth curves show treatment variability. Tumor volume data are shown for each mouse in the B16F10 model.

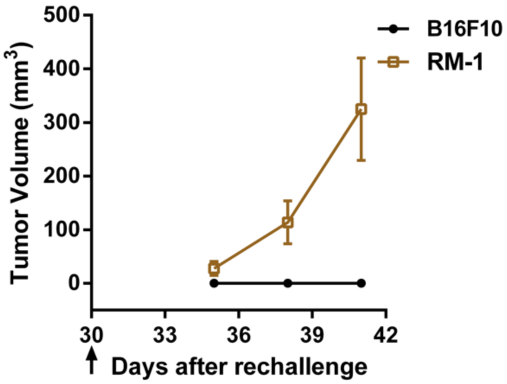

Supplementary Fig. 28. Cured mice resist melanoma rechallenge but not unrelated tumors. Tumor growth was assessed after rechallenge with different cell lines.

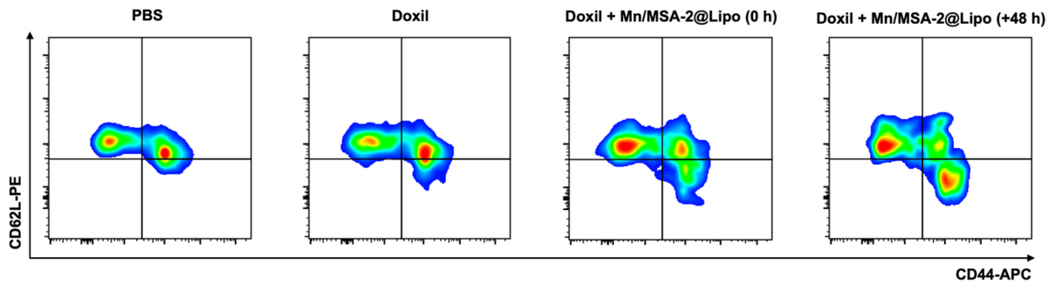

Supplementary Fig. 29. Effector memory CD8$^+$ T cells persist 40 days after therapy. Representative flow cytometry scatter plots showing splenic CD3$^+$CD8$^+$CD62L^{low}CD44^{hi} effector memory T cells (Tem) 40 days after completion of treatment.

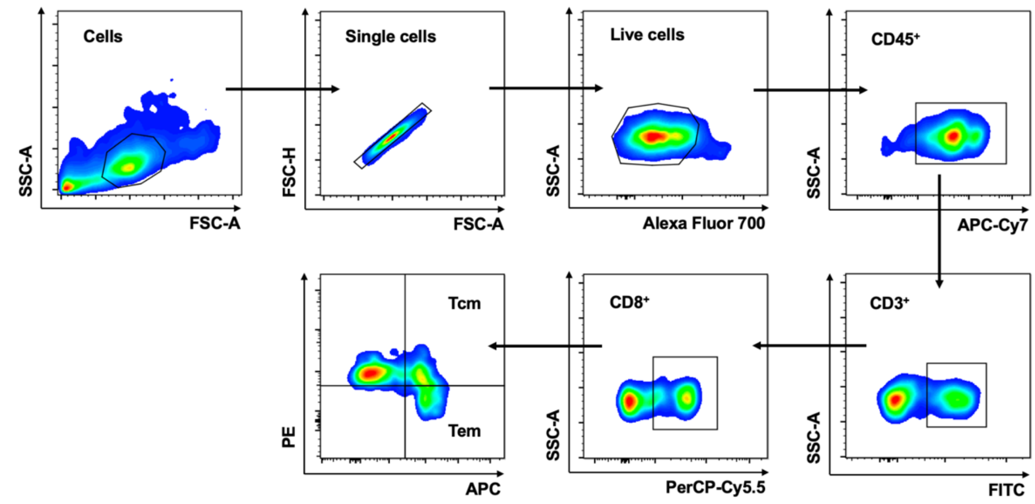

Supplementary Fig. 30. Flow cytometry gating strategy for identifying splenic Tem. $CD3^{+}CD8^{+}CD62L^{low}CD44^{hi}$ cells were defined as effector memory T cells.

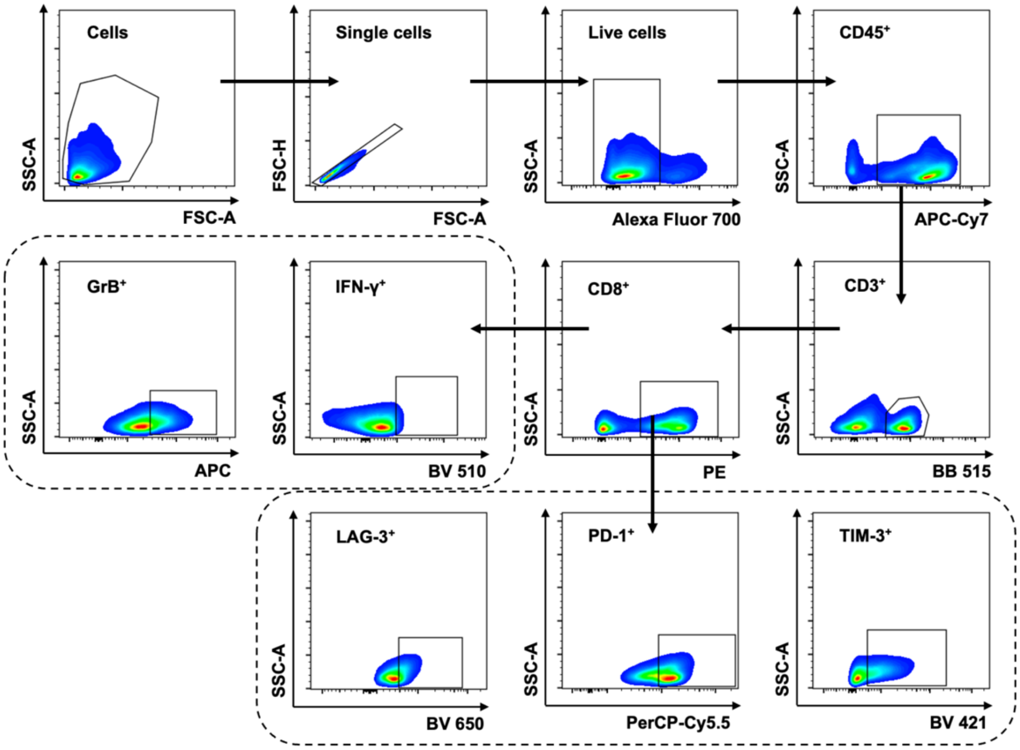

Supplementary Fig. 31. Gating strategy for tumor-infiltrating CD8$^+$ T cells. CD8$^+$ T cells were analyzed for function and exhaustion markers.

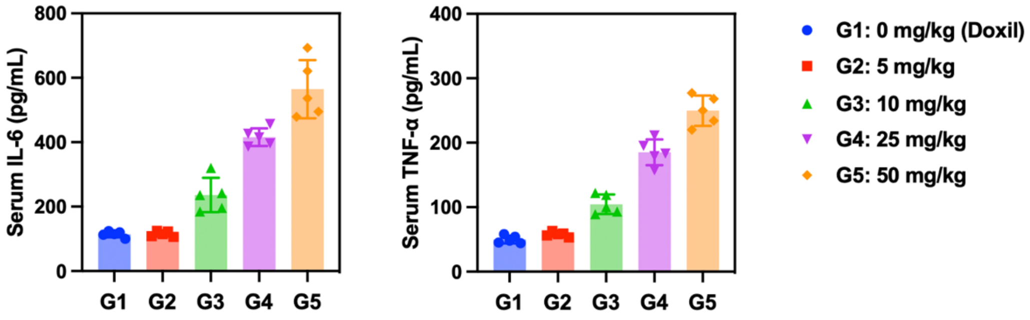

Supplementary Fig. 32. Therapeutic window identified based on systemic cytokine response. Serum cytokine levels of IL-6 and TNF-α measured 24 hours after Mn/MSA-2@Lipo administration at different doses (n = 5).

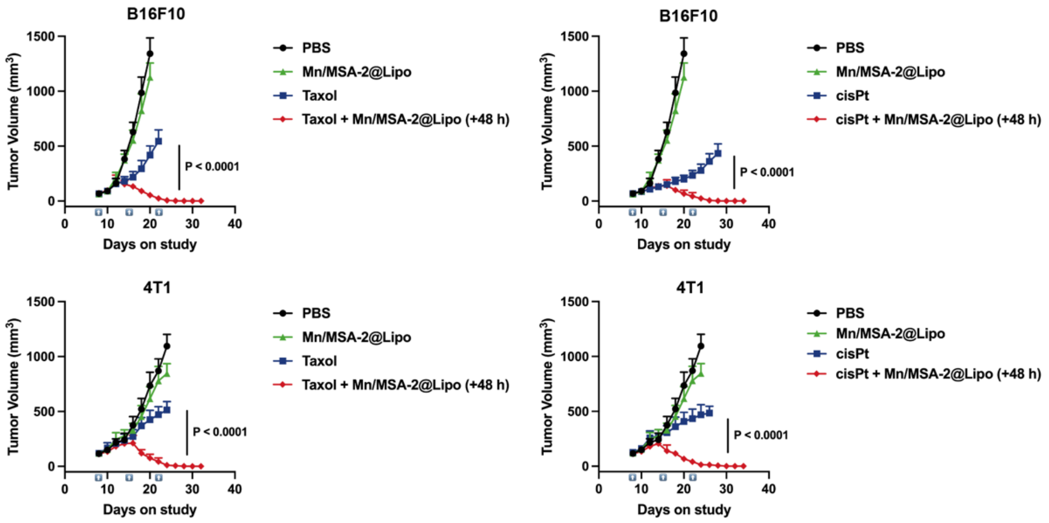

Supplementary Fig. 33. Combination with other chemotherapeutics maintains efficacy. Mn/MSA-2@Lipo combined with other frontline chemotherapeutics, including paclitaxel and cisplatin, using the same 48-hour interval, consistently achieved complete tumor ablation without recurrence.
